# Modelling of chromatographic and electrophoretic behaviour of imidazoline and alpha adrenergic receptors ligands under different acid-base conditions

**DOI:** 10.5599/admet.2278

**Published:** 2024-05-03

**Authors:** Slavica Oljacic, Mitja Križman, Marija Popovic-Nikolic, Irena Vovk, Katarina Nikolic, Danica Agbaba

**Affiliations:** 1 Department of Pharmaceutical Chemistry, Faculty of Pharmacy, University of Belgrade, Vojvode Stepe 450, 11000 Belgrade, Serbia; 2 Laboratory of Food Chemistry, National Institute of Chemistry, Hajdrihova 19, 1000 Ljubljana, Slovenia

**Keywords:** Quantitative structure-retention relationship, quantitative structure-mobility relationship, liquid chromatography, capillary electrophoresis

## Abstract

**Background and Purpose:**

The ligands of the imidazoline and α-adrenergic receptors are mainly imidazoline and guanidine derivatives, known as centrally-acting antihypertensives and compounds with potential use in various neurological disorders. The extent of their ionisation has a major influence on their behaviour in the different analytical systems. The main objective of this work was to compare the mechanism of chromatographic retention and electrophoretic mobility under acidic, neutral and basic conditions.

**Experimental Approach:**

Multiple Linear Regression and Partial Least Squares Regression were applied for the QSRR (quantitative structure-retention relationship) and QSMR (quantitative structure-mobility relationship) modelling and to select the most important molecular parameters describing the chromatographic and electrophoretic behaviour of the investigated compounds.

**Key Results:**

The most important molecular descriptors, such as the chemical composition of the compounds, lipophilicity, polarizability and molecular branching, in the selected QSRR models showed that an important insight into the retention behaviour can be derived from the 0D-, 1D- and 2D-descriptors. The electrophoretic mobility could be explained by 2D- and 3D-descriptors, which provide information on the molecular mass, size and complexity, as well as on the influence of charge transfer and electronic properties on the migration behaviour.

**Conclusion:**

All created QSRR/QSMR models met the stringent validation criteria and showed high potential in describing the chromatographic and electrophoretic behaviour of investigated compounds.

## Introduction

Imidazoline and α-adrenergic receptor ligands are mainly imidazoline and guanidine derivatives with different pharmacological effects and target tissues [1]. The pharmacological effects of these ligands are generally the results of interaction with three types of imidazoline receptors (I_1_-IR, I_2_-IR, and I_3_-IR) and α_2_-adrenoreceptors (α_2_-AR) [2,3]. Clonidine is a well-known first-generation centrally acting antihypertensive drug that lowers blood pressure by stimulating α_2_-AR and I_1_-IR. On the other hand, moxonidine and rilmenidine, which belong to the second generation of antihypertensives, act mainly at the I_1_-IR and produce fewer α_2_-adrenoceptor-mediated side effects [3-5]. Idazoxan and its analogs have a high affinity for the I_2_-IR whose involvement in different psychiatric disorders, opiate withdrawal, pain, and Parkinson’s and Alzheimer’s diseases has been investigated [6-8]. Efaroxan is able to induce insulin secretion from pancreatic β-cells by activating I_3_-imidazoline receptors (I_3_-IR) [9].

Considering the chemical structures of the above-mentioned ligands, the design of new compounds as drug candidates for the treatment of various cardiovascular or neurological diseases will be directed to compounds that contain one or more basic centers in their chemical scaffold and have the ability to ionize. For ionizable compounds, the extent of ionization together with lipophilicity is of great importance for their pharmacokinetic properties, such as absorption, distribution, metabolism and elimination (ADME), as well as for the drug-receptor interaction [10].

In addition, the extent of ionisation of compounds at different pH values has a strong effect on their behaviour in different separation systems, such as chromatographic and electrophoretic systems. As the degree of ionisation of basic compounds increases, their mobility in capillary electrophoresis increases, while the retention of unionised form of the analyte can be 10–20 times greater than that of the corresponding ionised form for a given mobile phase composition. Currently, high-performance liquid chromatography (HPLC) and capillary electrophoresis (CE) are very useful techniques for the rapid and efficient separation as well as examination of the individual imidazoline and α-adrenergic receptor ligands [11-14]. Therefore, accurate quantitative relationships between chromatographic retention or electrophoretic mobility and pH, which can be established through quantitative structure-retention relationships (QSRR) and quantitative structure-mobility relationships (QSMR) modelling can be very useful for predicting the retention behaviour and electrophoretic mobility of the new compounds, understanding the separation mechanism in a given analytical system and identifying important molecular descriptors [15]. In addition, the processes of drug action are believed to share many similarities with the processes underlying chromatographic separations, as the similar elementary intermolecular interactions are important for the behaviour of chemical compounds in both biological and chromategraphic environments [16]. Therefore, the chromatographic retention constant log *K*_w_ has been successfully used to evaluate the lipophilicity of compounds [17] as well as in correlation studies with various physicochemical and biological properties to establish appropriate QSPR (quantitative structure-property relationship) and QSAR (quantitative structure-activity relationship) models [18,19]. Chromatographic and electrophoretic data have also been successfully used for the pharmacological classification of compounds and prediction of their ADME properties [20]. HPLC was used to determine the effective permeability of 40 compounds (18 IRs/a-ARs ligands and 22 CNS drugs) using the parallel artificial membrane permeability assay (PAMPA) to predict brain penetration [21]. The electrophoretic behaviour of a compound is indicative of its acid-base properties. *i.e.*, p*K*_a_ values and molecular mass, since the charge-to-mass ratio of compounds largely determines their electrophoretic mobility. Thus, all these models, QSPR, QSRR, QSAR and QSMR, can provide us with valuable information about the activity, physicochemical and ADME properties of drug candidates and could, therefore, significantly reduce the number of drug candidates synthesised and tested in the early phase of drug discovery.

The main objective of this work was to investigate and compare the mechanism of chromatographic retention and electrophoretic mobility of 29 compounds with main or side effects to alpha adrenergic/imidazoline receptors. The study was performed at different ionization levels, including physiological pH, and applied to QSRR and QSMR models using multiple linear regression (MLR) and partial least squares regression (PLS). Rigorous validation criteria as proposed by Golbraikh and Tropsha [22,23] and Roy [24], were also applied as an essential part of QSRR/QSMR model development. The developed QSRR/QSMR models can be used for the fast and reliable prediction of the chromatographic and electrophoretic behavior of structurally related imidazoline and alpha adrenergic receptor ligands. In addition, the usefulness of chromatographic and electrophoretic data for predicting permeation across the blood-brain barrier (BBB) was also examined.

## Experimental

### Reagents and chemicals

Methanol (HPLC grade, 99.8 %) and glacial acetic acid (≥99.7 %) were purchased from J.T. Baker (Deventer, Netherlands). Ammonium acetate, sodium hydroxide, sodium dihydrogen phosphate, and disodium hydrogen phosphate of ACS reagent grade were purchased from Merck (Darmstadt, Germany). Boric acid (ACS reagent, 99.5 to 100.5 %) was purchased from Sigma-Aldrich (St. Louis, MO, USA). Ammonium hydroxide solution 30 % (analytical grade) was purchased from Carlo Erba (Milan, Italy). Aqueous solutions of background electrolytes and buffers for the preparation of mobile phases were prepared with water (HPLC grade).

Clonidine hydrochloride (≥98 %), moxonidine hydrochloride (≥98 %), guanfacine hydrochloride (≥98 %), brimonidine hydrochloride (≥98 %), efaroxan hydrochloride (≥98 %), idazoxan hydrochloride (≥98 %), rilmenidine hemifumarate (≥98 %), harmane (98 %), harmine hydrochloride (≥98 %), tizanidine hydrochloride (≥98 %), triamterene (≥99 %), clopamide (≥98 %), indapamide (≥99 %), naphazoline hydrochloride (≥98 %), xylometazoline hydrochloride (≥99 %), tetrahydrozoline hydrochloride (≥98 %), oxymetazoline hydrochloride (≥99 %), ephedrine hydrochloride (99 %), pseudoephedrine hydrochloride (99 %), maprotiline hydrochloride (>99 %), tamsulosin hydrochloride (≥98 %), mianserin hydrochloride (≥98 %), carvedilol (≥98 %), clozapine (≥98 %) and olanzapine (≥98 %) were purchased from Sigma–Aldrich (St. Louis, MO, USA). Tramazoline hydrochloride (≥98 %) and doxazosin mesilat (≥98 %) were obtained from Zdravlje-Actavis (Leskovac, Serbia). Amiloride hydrochloride (≥98 %) was kindly donated by Galenika (Belgrade, Serbia). Phenilephrine hydrochloride (98 %) was obtained from Ivančić i sinovi (Belgrade, Serbia).

### HPLC conditions

#### Solutions for HPLC analysis

The working solutions were prepared by dissolving the substances in methanol to obtain a concentration of 0.7 mg/mL for rilmenidine and 0.1 mg/mL for the other compounds.

#### HPLC equipment and working conditions

The HPLC working conditions are described in our previously published work [20]. HPLC analysis was performed using an Agilent Technologies 1200 (Wilmington, DE, USA) liquid chromatography system equipped with an online degasser, a binary pump, a column oven and a photodiode array detector. The data was recorded and analyzed using Agilent's ChemStation software (Chemstation for LC 3D system (Rev. B. 02. 01-SR2 [260]).

Separation was performed on the XTerra® RP18 column, 4.6×100 mm, particle size 3.5 μm (Waters Corporation, Milford, MA, USA) using three different constant ionic strength buffer solutions (*I* = 25 mmol/L) prepared at pH values 4.4, 7.4 and 9.1, as the aqueous component in the mobile phase while methanol added as an organic modifier. All measurements were performed at 25°C with a constant flow rate of 0.8 mL min^-1^ and UV detection in the range of 200-280 nm. The injection volume was 20 μL. Retention times were determined in the isocratic elution mode using at least six mobile phases of methanol/buffer (pH 4.4, 7.4 or 9.1) with a methanol concentration of 75-2 % depending on the retention properties of compounds. The retention factor *k* was calculated for each mobile phase composition according to Equation (1):



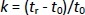

(1)


where *t*_r_ is the retention time of the tested compound and *t*_0_ is the dead volume measured with KNO_3_ as a non-retentive marker. The values corresponding to 100 % of the buffered eluent (log *k*_w_) were determined by extrapolation according to Equation (2):



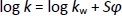

(2)


where log *k*_w_ is the intercept, *S* is the slope of the regression line, and *φ* is the volume fraction of the organic modifier in the mobile phase. Log *k*_w_ of the analyte at three different experimental conditions, methanol/buffer pH 4.4, methanol/buffer pH 7.4 and methanol/buffer pH 9.1 (log *k*_w4.4_, log *k*_w7.4_ and log *k*_w9.1_, respectively) were calculated for all investigated compounds and used for the QSRR analysis.

### Electrophoretic conditions

#### Solutions for CE analysis

Stock solutions of harman, harmine, clopamide, indapamide, rilmenidine, mianserin, doxazosin, carvedilol, clozapine, olanzapine and triamterene were prepared in methanol (*c* = 1 mg/mL) and diluted with water to a final concentration of 5.8 μg/mL for triamterene, 60 μg/mL for rilmenidine and 30 μg/mL for the other substances. Brimonidine was dissolved in 0.1 % formic acid, while the remaining substances were dissolved in water. Acetone 2 vol.% was used as electroosmotic flow (EOF) marker. Depending on UV responsiveness and solubility, samples were prepared at different concentrations, from 5.8 to 60 μg/mL.

#### CE equipment and working conditions

The CE working conditions are described in our previously published paper [20]. All experiments were performed using the SpectraPhoresis 500-capillary electrophoresis system (Spectra Physics Analytical, USA) with UV detector. Data were recorded and analyzed using ChromQuest software version 4.0 (Thermo Finnigan, USA).

The separations were performed with an uncoated fused capillary (total length 31.5 cm, 50 μm id, effective length 24 cm, Polymicro Technologies, USA) applying a voltage of 11 kV and at 25 °C. The samples were injected hydrodynamically and detection was performed in the 200-280 nm range. The new capillary was rinsed stepwise with 0.1 M NaOH (15 min), water (10 min) and a running buffer (10 min). Finally, a 10 min high voltage was applied through the background electrolyte-filled capillary to equalize the inner surface, stabilize the electroosmotic flow and ensure good reproducibility of injections from run to run. Background solutions with constant ionic strength (*I* = 25 mmol/L) were prepared at three different pH values by mixing the corresponding amounts of CH_3_COOH/NaOH, NaH_2_PO_4_/NaOH and H_3_BO_3_/NaOH for pH 4.4, 7.4 and 9.1, respectively. All compounds were injected in three replicates at each pH (4.4; 7.4 and 9.1). Between runs, the capillary was rinsed with the background electrolyte for 1 minute. The effective electrophoretic mobility, *μ*_eff_, of the analyte at three different pH values 4.4; 7.4 and 9.1 (*μ*_eff4.4_, *μ*_eff7.4_ and *μ*_eff9.1_, respectively) is expressed in cm^2^ / V s and was calculated using the Equation (3):



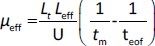

(3)


where *L*_t_ is the total length of the capillary (cm), *L*_eff_ is the effective length of the capillary (cm), *U* is the applied voltage (V), *t*_m_ is the migration time of the analyte (s) and *t*_eof_ is the migration time of the electroneutral EOF marker acetone (s). The calculated *μ*_eff_ values (*μ*_eff4.4_, *μ*_eff7.4_ and *μ*_eff9.1_) were used as dependent variables in QSMR analysis.

### Theory/calculation

#### Computational methods

Clonidine, moxonidine, tizanidine, brimonidine, rilmenidine, and tramazoline are cyclic guanidines or amidine structures that can exist in two main tautomeric forms (amino and imino). The stability of their amino and imino tautomers was investigated at the B3LYP/6–31G(d, p) level of density functional theory-(DFT) [25,26] using the Gaussian 98 program [27]. Based on the Self Consistent Field Energy calculated for neutral amino and imino tautomers of the compounds, it was shown that clonidine, moxonidine, tizanidine, brimonidine and tramazoline exist as more stable imino tautomers, while the predominant tautomeric form of rilmenidine is the amino form. The calculation of p*K*_a_ and the selection of the dominant molecule/cation species under experimental conditions (pH 4.4, 7.4 and 9.1) were performed for 29 α-adrenergic and imidazoline receptor ligands using the Marvin 5.5.1.0 ChemAxon program [28].

Structural optimization (selected tautomers and molecules/cations species at three different pH values) was performed using the B3LYP/3–21(d,p) levels of the DFT in the Gaussian 98 program [27]. Molecular descriptors were calculated for all investigated compounds in the programs Dragon [29], Gaussian 98 (B3LYP/3-21G(d,p) basis set) [27], Marvin 5.5.1.0 ChemAxon [28], and Chem3D Ultra 7.0.0 [30]. In addition, descriptors such as hardness (*η*), global softness, chemical potential (*μ*), electronegativity (*χ*) and electrophilicity index (*ω*), which have been extensively used to interpret various aspects of chemical bonding and reaction mechanism, were included in the set of calculated properties [31]. The partial least squares regression analysis started with 3445 computed descriptors. The descriptors with constant values were excluded from the modelling, as were descriptors with an intercorrelation greater than 0.996. For a pair of correlated descriptors, the one with the better correlation to Y was retained, while the other was excluded from the analysis. In the case of the MLR analysis, the cut-off value for the intercorrelation was set at 0.9 [32].

Quantitative structure-retention relationship and quantitative structure-mobility relationship modelling QSRR and QSMR modelling studies were performed for 29 imidazoline and α-adrenergic receptors ligands using PLS and stepwise MLR statistical methods at pH values of 4.4, 7.4 and 9.1 and on both HPLC and CE systems. An overview of the dataset was performed for each pH value and each chromatographic or electrophoretic system was examined in order to identify and exclude possible outliers before modelling. For this purpose, the scatter plots in the Simca P 12+ program were used [33], in which the pool of calculated molecular descriptors and the response variables are organised as an *X*-matrix. The scatter plot *t*_1_ *vs*. *t*_2_ is a window in *X*-space, showing how the *X* observations relate to each other, where *t*_1_ and *t*_2_ are scores (one vector for each model dimension) representing new variables computed as linear combinations of *X*. The score *t*_1_ (first component) explains the largest variation of the *X* space, followed by *t*_2_ and so on. For a two-dimensional score plot, the tolerance ellipse is presented based on Hotelling's T2. Observations that lie outside the ellipse are outliers. The *t*-*u* plot (*t*_1_ *vs. u*_1_) shows the relationship between *X* and *Y*. In addition, the degree of fit (a good fit corresponds to a small scatter around the straight line), indications of curvature and outliers can also be seen. The *u* score (one vector for each model dimension) is new variable summarizing *Y*, so as to maximize the correlation with the scores t. Based on the plots (*t*_1_ *vs*. *t*_2_ and *t*_1_ *vs*. *u*_1_), the remaining compounds were distributed among the training and test sets such that each chemical group (*i.e*., guanidine, 2-aminoimidazoline, 2-arylmethylimidazoline, phenylethylamine etc.) has a representative in the test set and the log *k*_w4.4_, log *k*_w7.4_, log *k*_w9.1_, *μ*_eff4.4_, *μ*_eff7.4_ and *μ*_eff9.1_ of the compounds in the test set are homogeneously distributed over the entire range of values of log *k*_w4.4_, log *k*_w 7.4_, log *k*_w9.1_, *μ*_eff4.4_, *μ*_eff7.4_ and *μ*_eff9.1_, respectively. The selected training and test sets contained at least 20 compounds used for model building and at least 5 compounds used for external validation. In order to perform a meaningful comparison between the different methods (MLR and PLS) used to develop the QSRR models, the same training and test set was used for each pH value. Therefore, six independent training and test sets were created, one for each dependent variable (log *k*_w4.4_, log *k*_w7.4_, log *k*_w9.1_, *μ*_eff4.4_, *μ*_eff7.4_ and *μ*_eff9._1). The predictive power and robustness of the models were estimated using the cross-validated parameter *R*^2^ (*Q*^2^), which was calculated from Equation (4):



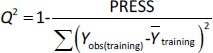

(4)


In Equation (4), PRESS is a parameter calculated using the leave-one-out (LOO) approach where each compound from the training set was deleted once and a new model was created with the remaining compounds and used to predict the Y-value of the deleted compound. The procedure was repeated until all compounds had been deleted once. For all created models, the squared sum of the differences between observed and LOO-predicted values (*e*(*i*)) (PRESS) was calculated according to Equation (5):



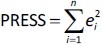

(5)


The models with *Q*^2^ ≥ 0.5 can be considered to have good predictive capability [34]. The root mean squared error of estimation (RMSEE) was calculated to characterize the predictive ability of the models for the compounds in the training set, Equations (6):



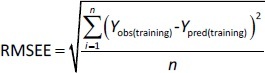

(6)


where *n* is the number of compounds in the training set, while *Y*_obs(training)_ and *Y*_pred(training)_ denote the experimental and predicted values for the compounds in the training set.

In addition to internal validation, the predictive power of all created models was estimated by external validation using the following parameters in Equations (7) and (8):



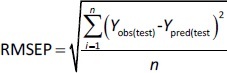

(7)


where RMSEP is the root mean square error of prediction, n is the number of compounds in the test set, while *Y*_obs(test)_ and *Y*_pred(test)_ are the experimental and predicted values for the compounds in the test set.



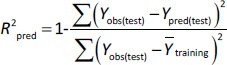

(8)


where *Y*_pred(test)_ and *Y*_obs(test)_ are the predicted and observed values of the dependent variables of the compounds in the test set, respectively, and *Y̅*_training_ indicates the mean values of the dependent variables of the compounds in the training set. For a predictive QSRR/QSMR model, the *R*^2^_pred_ value should be higher than 0.5 [35].

The rigorous external validation parameters proposed by Roy (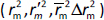) [24] and Golbraikh and Tropsha [22,23] were also applied to all created models and were used to select the most reliable models for the prediction of chromatographic and electrophoretic behaviour at each pH value.

According to Ojha and Roy [35], 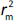 is validation metric calculated according to Equation (9) using observed (*y*-axis) and predicted (*x*-axis) values:



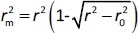

(9)


where *r*^2^ is the determination coefficient for the least squares regression line (with intercept) correlating observed (*y*-axis) and predicted (*x*-axis) values and 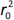 is the determination coefficient for the least squares regression line (without intercept) correlating observed (*y*-axis) and predicted (*x*-axis) values, 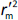 is validation metric calculated according to Equation (10) using observed (*x*-axis) and predicted (*y*-axis) values



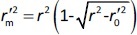

(10)


where 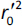 is the determination coefficient for the least squares regression line (without intercept) correlating observed (*x*-axis) and predicted (*y*-axis) values. 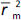 is average of 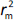 and 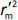, while 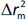 is the absolute difference between 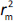 and 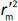.

Models were considered acceptable if they met all of the following conditions:

- the values of 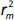 and 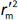 should be close to each other and 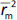 should be greater than 0.5;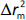 should be less than 0.2 [24].

According to criteria proposed by Golbraikh and Tropsha, following conditions should be achieved: (i) *Q*^2^ > 0.5; (ii) *R*^2^ > 0.6, (iii) [(*R*^2^ - *R*_0_^2^) / R^2^] < 0.1 or [(*R*^2^ - *R*'_0_^2^) / *R*^2^] < 0.1; (iv) 0.85 ≤ *k* ≤ 1.15 or 0.85 ≤ *k*' ≤ 1.15 where *Q*^2^ is the cross-validated correlation coefficient calculated for the training set, but all other criteria are calculated for the test set [22,23]. *R*_0_^2^ is the coefficient of determination (predicted versus observed values) for regressions through the origin. *R*'_0_^2^ is the coefficient of determination (observed versus predicted values) for regressions through the origin. *K* and *K*′ are slopes of regression lines through the origin.

### Multiple linear regression

Stepwise multiple linear regression was performed with the program STATISTICA [36]. A stepwise multiple linear regression procedure was used to select the molecular descriptors with the greatest influence on the dependent variables. The models were created stepwise according to the following procedure: identification of an initial model, then iterative „stepping“ in which descriptors were added or removed from the model based on the previously established stepping criteria (*F* to enter and *F* to remove) according to their statistical significance (*F*-test ), and finally termination of the search when no more stepping was possible or when a specified maximum number of steps was reached. Stepping criteria were set to a narrow range, and the predictive abilities of the models were tested by leave-one-out cross-validation and external test set prediction methods to avoid obtaining an overfitting model.

### Partial least squares regression

The PLS analysis was carried out using the SIMCA P+ 12.0 (soft independent modelling of class analogy) program [33]. The PLS approach is able to analyse data with noisy, collinear and even incomplete independent variables. In PLS modelling, the variable importance in the projection (VIP) parameter was used to assess the significance of the independent variables in the course of PLS model building. The VIP parameter represents a summary of the importance of each variable (*X*) for both the *Y* and *X* matrices. Molecular descriptors with VIP values greater than 1 are most important in explaining the regression model, those with 1.0 > VIP > 0.5 have a moderate influence, while the independent variables with VIP values less than 0.5 are not relevant for the model [33,37]. Therefore, during model generation, the descriptors with the lowest VIP values were successively removed from the model, while statistical parameters such as the squares of the multiple correlation coefficients *R*^2^, *Q*^2^ (a cross-validated version of *R*^2^), the root mean square error of estimation for the training set, and the root mean square error of prediction for the test set were calculated for each new PLS model and compared with the previous one. The procedure was repeated until the best models for the investigated experimental conditions (pH 4.4, 7.4 and 9.1) and analytical systems (HPLC and CE) were formed.

The response permutation test (*Y* scrambling), as a measure of model overfitting, was used to examine the statistical significance of the *R*^2^ and *Q*^2^ values and overfitting due to the chance correlation [37]. In this test, the *Y*-variables were randomly re-ordered 100 times while the *X*-matrix was remained unchanged. The PLS model was fitted to the permuted data and the new parameters *R*^2^, *Q*^2^ and VIP were calculated. All model selection steps were repeated with the scrambled *Y* response data. Regression lines were fitted through the *R*^2^ and *Q*^2^ values to obtain two separate intercepts. The values of the obtained intercepts for valid QSRR and QSMR models should be below 0.05 for the *Q*^2^ intercept and not above 0.4 for the *R*^2^ intercept [37].

## Results and discussion

The influence of the degree of ionization (under acidic, neutral and basic conditions) of 29 imidazoline and/or α-adrenergic receptor ligands (Figure S1, Supplementary material) on their retention in reversed-phase liquid chromatography and electrophoretic mobility in capillary electrophoresis was investigated. A pH value of 4.4 was chosen for the work in an acidic medium at which the majority of the tested compounds reached the highest degree of ionization, pH = p*K*_a_ - 2 (based on calculated p*K*_a_ values in [28] and experimentally determined p*K*_a_ values found in the literature, Table S1, Supplementary material). For operation in a neutral medium a pH value of 7.4 was used, which simulates physiological conditions, while a pH of 9.1 was chosen as a compromise where 9 compounds move together with the electroosmotic flow marker and the effective mobility is zero such as *e.g.* brimonidine (experimental p*K*_a_ determined by potentiometry 7.50), clozapine (experimental p*K*_a_ determined by HPLC 7.719 ± 0.015), doxazosine (experimental p*K*_a_ determined by voltammetry 6.89 ± 0.57), guanfacine (calculated basic p*K*_a_ 8.65), harman (experimental p*K*_a_ determined by HPLC 7.21), mianserin (experimental p*K*_a_ determined by potentiometry 7.40), moxonidine (experimental p*K*_a_ determined by HPLC 7.84), tizanidine (calculated p*K*_a_ 7.49) and triamterene (experimental p*K*_a_ determined by pH spectrophotometric method 7.16) (Tables S1 and S4, Supplementary material); about 1/3 of the compounds reach their mean mobility (pH close to the p*K*_a_ value, amiloride (experimental p*K*_a_ determined by potentiometry 8.72 ± 0.05), idazoxan (experimental p*K*_a_ determined by HPLC 9.04), phenylephrine (experimental p*K*_a_ determined by spectrophotometry 9.17), tamsulosin (calculated p*K*_a_ 9.28), clopamide (calculated acid p*K*_a_ 8.85), indapamide (experimental acid p*K*_a_ determined by potentiometry 8.8 ± 0.2) (Tables S1 and S4, Supplementary material) and the remaining compounds with p*K*_a_ values around and above 10 are still ionized to a high percentage, such as *e.g.* efaroxan (experimental p*K*_a_ determined by HPLC 10.02), ephedrine (experimental p*K*_a_ determined by spectrophotometry 9.65), maprotiline(experimental p*K*_a_ determined by potentiometry 10.45 ± 0.02), naphazoline (experimental p*K*_a_ determined by pH spectrophotometric method 10.81), oxymetazoline (experimental p*K*_a_ determined by pH spectrophotometric method 10.62), tetrahyrozoline (experimental p*K*_a_ determined by pH titration 10.51 ± 0.05), tramazoline (experimental p*K*_a_ determined by pH titration 10.66 ± 0.05)(Table S1 and S4, Supplementary material).

Two different linear statistical methods (MLR and PLS) were applied and compared for each experimental condition and examined systems in order to identify the most reliable models enabling accurate prediction of dependent variables.

### Quantitative structure-retention relationship modelling

#### Determination of log *k*_w_ values

The retention behaviour in HPLC system was monitored by calculating extrapolated retention factors log *k*_w_ corresponding to pure buffer as mobile phase. The log *k*_w_ values are considered to be better lipophilicity indices compared to the isocratic log *k*, as their values are of the same order of magnitude as the log *P* / log *D* octanol-water [38]. The values of log *k*_w_ (intercepts) and slopes (*S*) at all investigated pH values: 4.4, 7.4 and 9.1 are shown in Table S2 (Supplementary material). In an acidic environment (pH 4.4), the obtained log *k*_w_ values are in the range from 0.09 (phenilephrine) to 3.75 (carvedilol). Under the given conditions, only two compounds (clopamide and indapamide) are present in neutral (molecular) form, while the majority of the other compounds are completely ionized (over 99 %) [28]. This is also the reason for the lower log *k*_w_ values of the investigated compounds at pH 4.4 compared to pH 7.4 and 9.1, where the compounds are present in ionized form to a lesser extent or are predominantly non-ionized (pH 9.1). Therefore, as the pH increases, the lipophilicity of the compounds and, consequently, their log *k*_w_ values increase (Table S2, Supplementary material).

### Quantitative structure-retention relationship models

As a result of the QSRR studies applied to the log *k*_w_ data obtained at three pH values (log *k*_w4.4_, log *k*_w7.4_ and log *k*_w9.1_), six models were created: MLR-QSRR (log *k*_w4.4_), PLS-QSRR (log *k*_w4.4_), MLR-QSRR (log *k*_w7.4_), PLS-QSRR (log *k*_w7.4_), MLR-QSRR (log *k*_w9.1_), and PLS-QSRR (log *k*_w9.1_). The score plot of the first two components (*t*_1_ *vs. t*_2_) at each pH shows a uniform data distribution in all four quartiles. Tamsulosin is in group with oxymetazolin, clopamide, indapamide, carvedilol, doxazosin and oxymetazoline. These compounds are characterized by the highest descriptor values with tamsulosin lying outside or on the Hoteling T2 ellipse. Thus, tamsulosin was identified as an outlier in all chromatographic models.

For the generation of QSRR models at pH 4.4, 21 compounds were used in the training set, while the remaining 7 compounds (clopamide, clozapine, ephedrine, harman, mianserin, naphazoline, tizanidine) were used in the test set.

Using the stepwise MLR method, 4 descriptors with the greatest influence on the dependent variable log *k*_w4.4_ were selected, Equation (11):



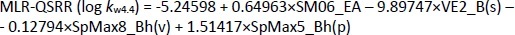

(11)


while statistically, the most significant PLS-QSRR model was formed with 6 descriptors, Equation (12):



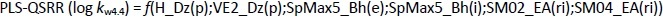

(12)


Better regression and validation parameters (Tables 1 and 2) were obtained for the PLS-QSRR(log *k*_w4.4_) model (*Q*^2^ = 0.865, RMSEP = 0.325, *F*= 57.524, *p* = 1.52E-08) compared to the MLR-QSRR(log *k*_w4.4_) model (*Q*^2^ = 0.764, RMSEP = 0.496, *F* = 23.502, *p* = 1.57E-06), so that the PLS-QSRR (log *k*_w4.4_) model was selected as optimal for the prediction of retention behaviour at pH 4.4.

**Table 1.** The most important statistical parameters obtained for MLR-QSRR and PLS-QSRR models

Model	*Q* ^2^	*R* ^2^ *Y*	*F*	*p*	RMSEE	RMSEP	*R* ^2^_intercept_	*Q*^2^_intercept_
MLR-QSRR (log *k*_w4.4_)	0.764	0.855	23.502	1.57×10^-6^	0.369	0.496		
PLS-QSRR (log *k*_w4.4_)	0.865	0.876	57.524	1.52×10^-8^	0.341	0.325	-0.0969	-0.210
MLR-QSRR (log *k*_w7.4_)	0.846	0.907	54.995	5.82×10^-9^	0.365	0.446		
PLS-QSRR (log *k*_w7.4_)	0.883	0.903	28.978	3.76×10^-7^	0.371	0.378	0.0091	-0.279
MLR-QSRR (log *k*_w9.1_)	0.931	0.953	113.538	1.91×10^-11^	0.226	0.353		
PLS-QSRR (log *k*_w9.1_)	0.915	0.924	96.874	2.32×10^-10^	0.286	0.359	-0.0781	-0.224

**Table 2.** External validation parameters for QSRR models, based on Golbraikh, Tropsha, and Roy [22-24]

Model	*R* ^2^	*R*_0_^2^	(*R*^2^-*R*_0_^2^)/*R*^2^	*k*	*R*'_0_^2^	(*R*^2^-*R*'_0_^2^)/*R*^2^	*k*'	*R* ^2^_pred_	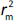	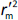	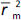	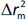
MLR-QSRR (log*k*_w4.4_)	0.635	0.618	0.028	0.981	0.578	0.090	0.950	0.617	0.551	0.484	0.517	0.067
PLS-QSRR (log*k*_w4.4_)	0.835	0.835	0.000	1.003	0.806	0.035	0.968	0.835	0.830	0.693	0.762	0.138
MLR-QSRR (log*k*_w7.4_)	0.829	0.782	0.056	0.943	0.827	0.002	1.015	0.766	0.650	0.793	0.722	0.144
PLS-QSRR (log*k*_w7.4_)	0.854	0.839	0.018	0.962	0.854	0.000	1.006	0.832	0.748	0.843	0.796	0.095
MLR-QSRR (log*k*_w9.1_)	0.910	0.877	0.036	0.960	0.802	0.118	1.022	0.872	0.745	0.611	0.678	0.134
PLS-QSRR (log*k*_w9.1_)	0.874	0.872	0.002	0.960	0.868	0.007	1.022	0.868	0.838	0.805	0.822	0.033

The QSRR models at pH 7.4 were created with a training set of 21 compounds, while the remaining 7 compounds (clonidine, ephedrine, maprotiline, olanzapine, tizanidine, triamterene, xylometazoline) were used in the test set. The best MLR-QSRR model was obtained with 3 descriptors, Equation (13):



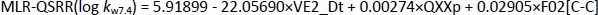

(13)


The PLS analysis resulted in the model with five important descriptors, Equation (14):



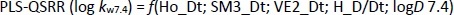

(14)


Although the MLR-QSRR (log *k*_w7.4_) model has statistically more significant values for *F*, *p* and *R*^2^ than the PLS-QSRR (log *k*_w7.4_) model, better validation parameters (*Q*^2^ = 0.883; *R*^2^_test_=0.854; RMSEP = 0.378) for the PLS model compared to the MLR model ((*Q*^2^ = 0.846; *R*^2^_test_ = 0.829; RMSEP = 0.446) (Tables 1 and 2) were allocateed this regression model as more reliable for predicting the retention behaviour of related compounds at pH 7.4.

At pH 9.1, the test set consisted of 7 compounds (clozapine, efaroxan, ephedrine, maprotiline, tizanidine, triamterene, xylometazoline) and a training set of 21 compounds.

The most significant MLR model describing the retention behaviour at pH 9.1 consists of 3 molecular descriptors, Equation (15):



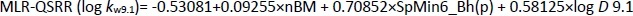

(15)


The statistically best PLS model was formed with 5 descriptors, Equation (16):



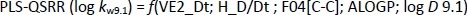

(16)


The analysis of the calculated statistical parameters (Tables 1 and 2) obtained for the created models at pH 9.1, suggests that the MLR-QSRR (log *k*_w9.1_) model can be selected as an optimal and reliable model for predicting the retention behaviour of α-adrenergic and imidazoline receptor ligands in an alkaline environment.

In addition, it should be emphasised that all created MLR-QSRR and PLS-QSRR models meet the external validation criteria proposed by Golbraikh, Tropsha and Roy (Table 2) [22-24].

**Figure 1.**
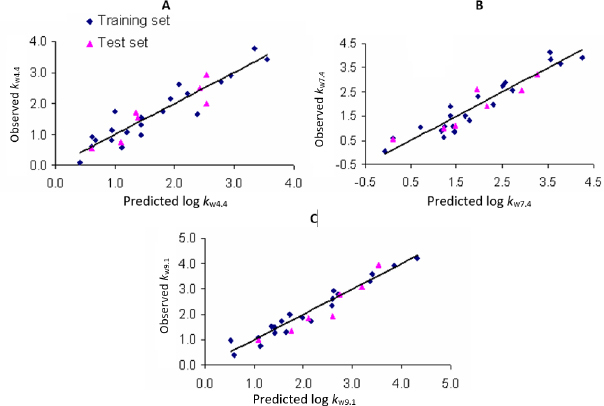
Observed *vs*. predicted values in selected QSRR models: A - PLS-QSRR (log *k*_w4.4_); B - PLS-QSRR (log *k*_w7.4_); C - MLR-QSRR (log *k*_w9.1_)

Plots of observed *vs*. predicted values for compounds in the training and test sets in selected QSRR models are depicted in Figure 1. The values of molecular descriptors in selected QSRR models are listed in Table S3.

### Quantitative structure-mobility relationship modelling

#### Determination of effective electrophoretic mobility

The effective electrophoretic mobility of the investigated compounds was determined in the presence of acetone as a neutral marker, and the results obtained under the examined conditions (pH 4.4, 7.4 and 9.1) are presented in Table S4 (Supplementary Material). At pH 4.4, 27 compounds that move faster than the neutral marker are in the form of cations and reach their maximum effective mobility, while the effective mobility of clopamide and indapamide, which are in neutral form under the studied conditions, is zero. The range of the obtained *μ*_eff4.4_ values is between 0 (indapamide, clopamide) and 28.19×10^-5^ cm^2^ / V s (harmane). At a physiological pH of 7.4, compounds with p*K*_a_ values around 7.4 have reached their intermediate mobility, indapamide and clopamide co-migrate with the EOF marker (*μ*_eff_ = 0), and compounds with a p*K*_a_ value above 10 (Table S1, Supplementary material), a high percentage of which are in the form of cations under the given conditions, have maintained an effective mobility similar to that in an acidic environment. The resulting range of effective mobility is between 0 and 24.18×10^-5^ cm^2^ / V s (naphazoline). In an alkaline environment at pH 9.1, negatively charged ions of clopamide and indapamide migrate to the cathode more slowly than EOF markers and exhibit negative values of effective mobility. Uncharged compounds migrate together with the EOF marker and their mobility is zero, while the derivatives of 2-methylimidazoline with p*K*_a_ values above 10 maintain a high degree of mobility (*μ*_eff_ = 25.05×10^-5^ cm^2^ / V s, tetrahydrozoline) (Table S1 and Table S4).

#### Quantitative structure-mobility relationship models

As a result of the QSMR studies applied to the *μ*_eff_ data obtained at three pH values (*μ*_eff4.4_, *μ*_eff7.4_ and *μ*_eff9.1_), six models were created: MLR-QSMR (*μ*_eff4.4_), PLS-QSMR (*μ*_eff4.4_), MLR-QSMR (*μ*_eff7.4_), PLS-QSMR (*μ*_eff7.4_), MLR-QSMR (*μ*_eff9.1_), and PLS-QSMR (*μ*_eff9.1_).

The QSMR models, which describe the mobility of the investigated compounds at a pH of 4.4, were created with 20 compounds in the training set. Three compounds, one with the highest electrophoretic mobility (olanzapine *μ*_eff4.4_ = 33.632 ± 0.036 10^-5^ cm^2^ / V s) and two with the lowest electrophoretic mobility (clopamide *μ*_eff4.4_ = 0.000 ± 0.000 and indapamide *μ*_eff4.4_ = 0.000 ± 0.000) clearly showed a significant deviation from the regression line on the *t*_1_ *vs. u*_1_ scater plot and were therefore identified as outliers. The remaining 6 compounds (amiloride, carvedilol, ephedrine, mianserin, tizanidine and xylometazoline) were used for external validation.

The most significant MLR-QSMR model at pH 4.4 was formed with 4 descriptors, Equation (17):



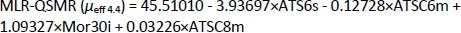

(17)


The PLS analysis also selected the model with the four descriptors that showed the greatest influence on the dependent variable *μ*_eff 4.4_, Equation (18)



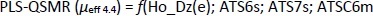

(18)


According to the better statistical parameters of the PLS-QSMR model (*Q*^2^ = 0.931, *F* = 115.508, *p* = 1.28×10^-10^, *R*^2^_test_ = 0.897 and RMSEP = 0.984) compared to the MLR-QSMR model (*Q*^2^ = 0.880, *F* = 64.338, *p* = 2.92×10^-9^, *R*^2^_test_ = 0.876 and RMSEP = 1.063) (Tables 3 and 4), the PLS-QSMR model can be selected as the optimal and reliable model for predicting the electrophoretic mobility of related compounds at pH 4.4.

**Table 3.** The most important statistical parameters obtained for MLR-QSMR and PLS-QSMR models

Model	*Q* ^2^	*R* ^2^	*F*	*p*	RMSEE	RMSEP	*R* ^2^_intercept_	*Q* ^2^_intercept_
MLR-QSMR (*μ*_eff 4.4_)	0.880	0.945	64.338	2.92E-09	0.739	1.063		
PLS-QSMR (*μ*_eff 4.4_)	0.931	0.939	115.508	1.28E-10	0.776	0.984	-0.111	-0.228
MLR-QSMR (*μ*_eff 7.4_)	0.858	0.932	51.412	1.4E-08	1.569	2.216		
PLS-QSMR (*μ*_eff 7.4_)	0.855	0.870	50.098	7.47E-08	2.170	3.244	-0.0245	-0.225
MLR-QSMR (*μ*_eff 9.1_)	0.891	0.936	58.736	2.32E-09	2.640	3.794		
PLS-QSMR (*μ*_eff 9.1_)	0.882	0.916	29.464	3.35E-07	3.033	4.290	0.046	-0.329

**Table 4.** External validation parameters for QSMR models, based on Golbraikh, Tropsha, and Roy [22-24]

Model	*R* ^2^	*R*_0_^2^	(*R*^2^-*R*_0_^2^)/*R*^2^	*k*	*R*'_0_^2^	(*R*^2^-*R*'_0_^2^)/*R*^2^	*k*'	*R* ^2^_pred_	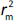	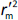	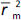	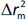
MLR-QSMR (*μ*_eff 4.4_)	0.876	0.862	0.016	0.999	0.876	0.000	0.999	0.869	0.772	0.865	0.819	0.094
PLS QSMR (*μ*_eff 4.4_)	0.893	0.882	0.013	1.002	0.893	0.000	0.996	0.888	0.798	0.888	0.843	0.089
MLR-QSMR (*μ*_eff 7.4_)	0.788	0.771	0.022	1.003	0.781	0.009	0.980	0.799	0.685	0.722	0.703	0.037
PLS-QSMR (*μ*_eff 7.4_)	0.686	0.563	0.178	0.938	-0.818	2.192	1.030	0.570	0.446	-0.155	0.145	0.601
MLR-QSMR (*μ*_eff 9.1_)	0.879	0.860	0.022	0.843	0.814	0.075	1.096	0.802	0.758	0.654	0.706	0.104
PLS-QSMR (*μ*_eff 9.1_)	0.873	0.834	0.045	0.813	0.750	0.141	1.118	0.747	0.701	0.567	0.634	0.134

QSMR models at pH 7.4 were formed with 20 compounds in the training set, while 5 compounds (ephedrine, olanzapine, oxymetazoline, tamsulosin and tizanidine) were retained in the test set. Considering the fact that the investigated data sets are very different in their acid-base properties, the resulting differences in effective electrophoretic mobility are significant (Table S1, Supplementary material).

Clopamide and indapamide, which carry a weakly acidic sulfonamide group, are still predominantly unionized at pH 7.4 and move together with the electroosmotic flow, resulting in an electrophoretic mobility of zero.

A very low effective electrophoretic mobility is also obtained for triamterene (*μ*_eff7.4_ = 1.140±0.053××10^-5^ cm^2^ / V s) as a weak base and guanfacine (*μ*_eff7.4_ = 5.083 ± 0.025×10^-5^ cm^2^ / V s). The Y-values of these compounds in relation to the calculated X-values showed a significant deviation in the scatter plot *t*_1_ *vs. u*_1_ and were identified as outliers. The most significant MLR-QSMR model is composed of 4 descriptors, Equation (19):



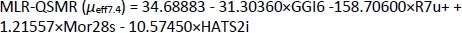

(19)


Using PLS analysis, a model was constructed with 5 molecular descriptors that significantly affect the electrophoretic mobility of the studied compounds at pH 7.4, Equation (19):



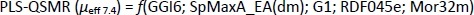

(20)


For the MLR-QSMR model, lower values for RMSEE and RMSEP, slightly better parameters *Q*^2^ and *F* value as well as significantly higher values for *R*^2^_trening_ and *R*^2^_test_ were obtained compared to the PLS-QSMR model (Tables 3 and 4). In addition, the PLS-QSMR model did not meet external validation criteria established by Golbraikh and Tropsha [22] [(*R*^2^ - 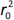) / *R*^2^ = 0.178 > 0.1; *R*'_0_^2^= -0.818; (*R*^2^- *R*'_0_^2^ ) / *R*^2^ = 2.192 > 0.1] (Table 4); and Roy [23,24] [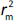 =0.446 and 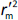 = -0.155 are not closed; 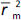 = 0.145 < 0.5; 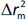
= 0.601 > 0.2] (Table 4) indicating that the MLR-QSMR regression equation can be reliably applied to predict the electrophoretic mobility of related compounds at pH 7.4.

At a pH of 9.1, nine compounds migrate together with EOF and have an effective mobility of 0. Five compounds with an effective mobility of zero remain in the training set, while four compounds move to the test set taking into account that all chemical clusters have a representative in the test set. Since all created models were unable to predict the mobility of harman and mianserin (*μ*_eff_ = 0) and taking into account that these two compounds had the greatest deviation on the t_1_ *vs*. u_1_ plot, among others from the training set with zero mobility, these two compounds were excluded from further analysis.

QSMR models at pH 9.1 were created with 21 compounds in the training set, while 6 compounds (doxazosin, ephedrine, idazoxan, olanzapine, tizanidine, xylometazoline) were used for external validation.

By MLR analysis, four significant molecular descriptors were selected, Equation (21):



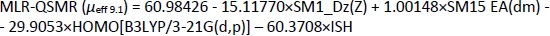

(21)


While the statistically best PLS model was formed with 5 important molecular parameters, Equation (22):



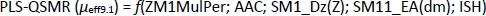

(22)


Appropriate validation criteria were met for both MLR-QSMR/PLS-QSMR models created (Tables 3 and 4), with the exception of the value (*R*^2^-*R*'_0_^2^)/*R*^2^ 0.141 > 0.1 of the PLS-QSMR (*μ*_eff9.1_) model (Table 4). Due to the higher values of *Q*^2^, *F*, *R*^2^_trening_, *R*^2^_test_ (*Q*^2^ = 0.891, *F* = 58.736, *R*^2^_traning_ = 0.936 and *R*^2^_test_ =0.879) for the MLR model than for the PLS model (*Q*^2^ = 0.882, *F* = 29.464, *R*^2^_trening_ = 0.916 and *R*^2^_test_ = 0.873) and the lower RMSEE and RMSEP (RMSEE = 2.640 and RMSEP = 3.794) for the MLR model than for the PLS model (RMSEE=3.033 and RMSEP = 4.290), the MLR-QSMR model: MLR-QSMR (*μ*_eff9.1_) = *ƒ*(SM1_Dz (Z); SM15_EA (dm); HOMO [B3LYP/3--21G (d, p)]; ISH) was selected as the optimal model for predicting the effective mobility of related compounds at a given pH of 9.1.

**Figure 2.**
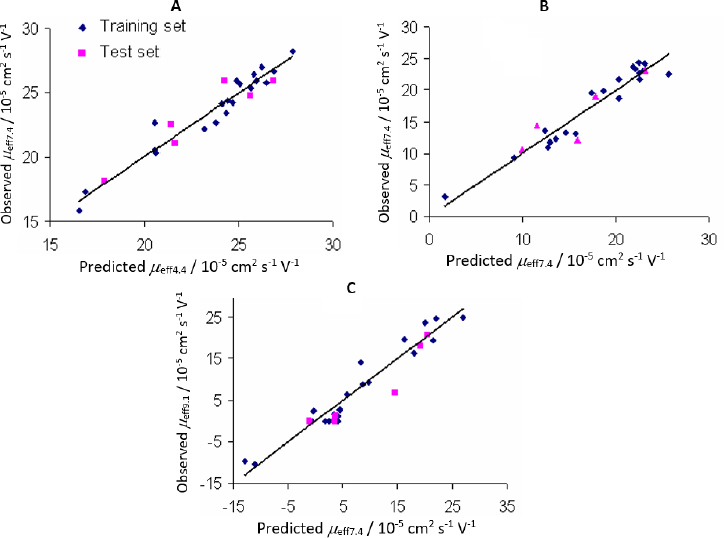
Observed *vs*. predicted values in selected QSMR models: A - PLS-QSMR (*μ*_eff4.4_); B - MLR-QSMR (*μ*_eff7.4_); C - MLR-QSMR (*μ*_eff9.1_)

Plots of observed *vs.* predicted values for compounds in the training and test sets of the selected QSMR models are depicted in Figure 2.

It is interesting to note that in Figure 2B, which shows the correlation between the calculated and predicted values of the QSMR model at pH 7.4, two clusters of data can be observed. The upper cluster includes compounds whose experimentally determined effective mobility is greater than 19×10^-5^ cm^2^ / V s and experimental p*K*_a_ value is greater than 8, and the lower cluster includes compounds with experimental effective mobility of less than 15×10^-5^ cm^2^ / V s and p*K*_a_ value less than 8 (Table S1 and S4, Supplementary material). The selected MLR-QSMR model could be used for a rough prediction of the p*K_a_* values of new imidazoline analogs.

The values of the molecular descriptors in the selected QSMR models are listed in Table S5 (Supplementary Material).

### Interpretation and comparison of selected quantitative structure-retention relationship and quantitative structure-mobility relationship models

Based on the presented statistical results (Tables 1-4) obtained after internal and rigorous external validation, optimal QSRR and QSMR models were selected for each investigated pH value. The classes of descriptors in the selected models and their descriptions are presented in Tables 5 and 6.

**Table 5.** The most important descriptors in selected QSRR models

Selected descriptors	Description	Class of descriptors
H_Dz(p)	Harary-like index from Barysz matrix weighted by polarizability	2D matrix-based descriptors
VE2_Dz(p)	Average coefficient of the last eigenvector from Barysz matrix weighted by polarizability	2D matrix-based descriptors
SpMax5_Bh(e)	Largest eigenvalue n. 5 of Burden matrix weighted by Sanderson electronegativity	Burden eigenvalues
SpMax5_Bh(i)	Largest eigenvalue n. 5 of Burden matrix weighted by ionization potential	Burden eigenvalues
SM02_EA(ri)	Spectral moments of order 2 from edge adjacency mat. weighted by resonance integral	Edge adjacency indices
SM04_EA(ri)	Spectral moments of order 4 from edge adjacency mat. weighted by resonance integral	Edge adjacency indices
Ho_Dt	Hosoya-like index (log function) from detour matrix	2D matrix-based descriptors
SM3_Dt	Spectral moment of order 3 from detour matrix	2D matrix-based descriptors
VE2_Dt	Average coefficient of the last eigenvector from detour matrix	2D matrix-based descriptors
H_D/Dt	Harary-like index from distance/detour matrix	2D matrix-based descriptors
log *D*_7.4_	Logarithm of distribution coefficient at pH 7.4	Molecular properties
nBM	Number of multiple bonds	Constitutional indices
SpMin6_Bh(p)	Smallest eigenvalue n. 6 of Burden matrix weighted by polarizability	Burden eigenvalues
log *D*_9.1_	Logarithm of distribution coefficient at pH 9.1	Molecular properties

**Table 6.** The most important descriptors in selected QSMR models

Selected descriptors	Description	Class of descriptors
Ho_Dz(e)	Hosoya-like index (log function) from Barysz matrix weighted by Sanderson electronegativity	2D matrix-based descriptors
ATS6s	Broto-Moreau autocorrelation of lag 6 (log function) weighted by I-state	2D autocorrelations
ATS7s	Broto-Moreau autocorrelation of lag 7 (log function) weighted by I-state	2D autocorrelations
ATSC6m	Centred Broto-Moreau autocorrelation of lag 6 weighted by mass	2D autocorrelations
GGI6	Topological charge index of order 6	2D autocorrelations
R7u+	R maximal autocorrelation of lag 7 / unweighted	GETAWAY descriptors
Mor28s	Signal 28 / weighted by I-state	3D-MoRSE descriptors
HATS2i	leverage-weighted autocorrelation of lag 2 / weighted by ionization potential	GETAWAY descriptors
HOMO [B3LYP/3-21G(d,p)]	Highest occupied molecular orbital	Quantum chemical
SM1_Dz(Z)	Spectral moment of order 1 from Barysz matrix weighted by atomic number	2D matrix-based descriptors
ISH	Standardized information content on the leverage equality	GETAWAY descriptors
SM15_EA(*dm*)	Spectral moment of order 15 from edge adjacency mat. weighted by dipole moment	Edge adjacency indices

The descriptors selected for the optimal PLS-QSRR (log *k*_w4.4_) model belong to three different descriptor classes (Table 5), such as 2D matrix-based descriptors (H_Dz(p), VE2_Dz(p)), Burden eigenvalues (SpMax5_Bh(e), SpMax5_Bh(i)) and Edge adjacency indices (SM02_EA(ri), SM04_EA(ri)).

**Figure 3.**
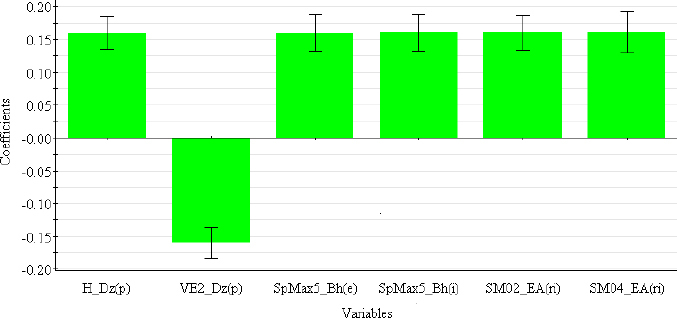
Coefficient plot of descriptors in PLS-QSRR (log *k*_w4.4)_ model.

The coefficient plot (Figure 3) shows that all descriptors have a positive influence, with the exception of VE2_Dz(p), which has a negative influence on the log *k*_w4.4_ value.

2D matrix-based descriptors are topological indices computed by applying a set of basic algebraic operators to different graph-theoretical matrices representing an H-depleted molecular graph [29]. Depending on which matrix they are derived from (detour matrix Dt, Barysz matrices (Dz(w)), *etc*.), they can be divided into different sub-blocks. H_Dz(p) (Harary-like index from Barysz matrix weighted by polarizability) and VE2_Dz(p) (average coefficient of the last eigenvector from the Barysz matrix weighted by polarizability) are derived from the Barysz matrices. Barysz matrices (Dz(w)) are weighted distance matrices that take into account the presence of heteroatoms and multiple bonds in the molecule. Polarizability is an important atomic property included in both the VE2_Dz(p) and H_Dz(p) descriptors, which differ in their influence on log *k*_w4.4_. Higher values of VE2_Dz(p) are associated with less lipophilic compounds in the studied group that have lower log *k*_w4.4_ values (ephedrine, rilmenidine), while lower values are seen for the structures of carvedilol, clozapine and maprotiline with the highest log *k*_w4.4_ values. The opposite effect can be observed for H_Dz(p). SpMax5_Bh(e) and SpMax5_Bh(i) are the largest eigenvalue *n*. 5 of the Burden matrix weighted by Sanderson electronegativity and ionization potential, respectively. Burden eigenvalues descriptors were originally proposed for searching for chemical similarity/diversity in large databases. They are calculated from the Burden matrix as an ordered sequence of the largest positive and smallest negative eigenvalues that reflect relevant aspects of molecular structure and can be useful for similarity searches [29,39].

SM02_EA(ri) and SM04_EA(ri) (spectral moments of order 2 and 4 from edge adjacency mat. weighted by resonance integral) belong to the Edge adjacency indices descriptors. These indices are based on the edge adjacency matrix of a graph. They encode information about the connectivity between graph edges, taking into account bonds between non-hydrogen atom pairs. Dragon calculates the Edge adjacency matrices using the following bond properties: edge degree (*ed*), dipole moment (*dm*), conventional bond order (*bo*) and parameters related to the resonance integral (*ri*). The spectral moment represents the linear combination of different structural fragments in the graph [29,40,41].

In contrast to chromatographic behaviour, electrophoretic mobility in an acidic medium is primarily determined by 2D autocorrelation descriptors (ATS6s, ATS7s, ATSC6m) (Table 6), which describe how the observed property (in this case, the intrinsic state in ATS6s and ATS7s and the mass in ATSC6m) is distributed along the topological structure.

**Figure 4.**
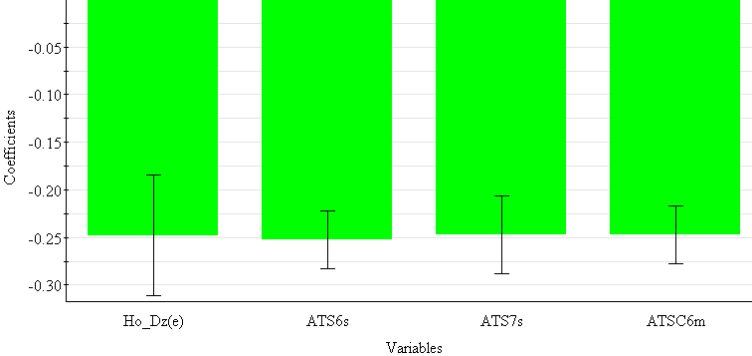
Coefficient plot of descriptors in PLS-QSMR (*μ*_eff4.4_) model

This class of descriptors represents the homogeneity of the molecular structure and can be weighted by the intrinsic state (*s*), the Sanderson electronegativity (*e*), the atomic mass (*m*) and the van der Waals volume (*v*) [42]. H0_Dz(e) (Hosoya-like index (log function) from the Barysz matrix, weighted by the Sanderson electronegativity) is 2D-matrix-based descriptor and represents a measure of molecular size and branching [43]. From the coefficient plot (Figure 4), It can be concluded that all selected descriptors have a negative influence on the *μ*_eff4.4_ values.

At a physiological pH of 7.4, retention behaviour is a function of 2D matrix-based descriptors and molecular properties (Table 5).

**Figure 5.**
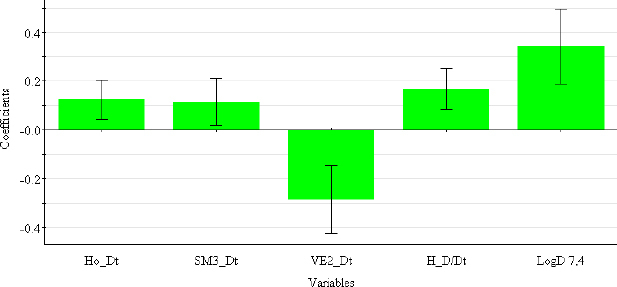
Coefficient plot of descriptors in PLS-QSRR (log *k*_w7.4_) model

Selected 2D matrix-based descriptors are derived from the detour matrix (Ho_Dt, SM3_Dt, VE2_Dt) and the distance/detour matrix (H_D/Dt), in contrast to the 2D matrix-based descriptors at pH 4.4. Ho_Dt is a Hosoya-like index (log function) from the detour matrix and represents a molecular descriptor that depends on the size of the molecule as well as its voluminosity [43-45]. Molecules with a simple structure, such as ephedrine and phenilephrine have the lowest value of Ho_Dt, while complex structures, such as those found in doxazosin and tetracyclic (mianserin) and tricyclic compounds (carvedilol, clozapine) have the highest value of this descriptor. H_D/Dt (Harary-like index from distance/detour matrix) is also a parameter sensitive to steric effects and whose value increases with increasing molecule size and branching [46]. Both descriptors are positively correlated with the log *k*_w7.4_ data (Figure 5.), which means that more lipophilic compounds are characterised by a higher value of this descriptor.

The logarithm of distribution coefficient at pH 7.4, log *D*_7.4_ [28], is a measure of the lipophilicity of compounds at pH 7.4 and indicates the importance of considering the presence of ionic species when evaluating the lipophilicity of compounds at pH 7.4. The selected model shows that calculations of all proposed descriptors allow a more reliable prediction of compound lipophilicity and that the design of compounds with the desired properties is influenced by the presence of groups responsible for steric effects and the size of the molecule.

In capillary electrophoresis, the electrophoretic mobility of investigated compounds at pH 7.4 can be discussed on the basis of selected 2D autocorrelations (GGI6), GETAWEY and 3D-MoRSE descriptors (Table 6). In contrast to the PLS-QSMR (μ_eff4.4_) model, in which the class of 2D autocorrelation descriptors dominates, the 3D descriptors appear to be more important in the selected MLR-QSMR (μ_eff7.4_) model.

GETAWAY descriptors combine the 3D-molecular geometry provided by the molecular influence matrix and atomic relatedness through molecular topology with chemical information by using different atomic weights (atomic mass, polarizability, van der Waals volume and electronegativity) together with unit weights [47]. GETAWAYs (HATS2i, R7u+) are geometric descriptors that are sensitive to molecular branching and cyclicity, encode information about the effective position of substituents and fragments in molecular space, and may be suitable to describe differences in congeneric molecular series [29]. Equation 19 shows that both descriptors (HATS2i, R7u+) have a negative influence on the *μ*_eff7.4_ values.

3D-MoRSE descriptors (Mor28s) represent the 3D structure of molecules based on electron diffraction [48]. Mor28s is a signal 28 / weighted by I-state and, in contrast to the GETAWAY descriptors, has a positive influence on the *μ*_eff7.4_ parameter (Equation 19).

All molecular descriptors selected with the MLR-QSRR (log *k*_w9.1_) model (Equation (15)) have a positive influence on log *k*_w9.1_ and belong to constitutional indices, Burden eigenvalues and molecular properties classes of descriptors (Table 5).

Constitutional descriptors such as the selected nBM (number of multiple bonds), which counts a molecule's double, triple and aromatic bonds, are the simplest and most commonly used descriptors. They represent the chemical composition of a compound without information about the molecular geometry or the connectivity of the atoms [29]. SpMin6_Bh(p) is the smallest eigenvalue n.6 of the Burden matrix weighted by polarizability and belongs to the class of Burden eigenvalue descriptors. The lipophilicity of compounds at pH 9.1 (log *D*_9.1_) [28] appears again as an important molecular property considering all forms of the molecule (ionised and unionised), and its high positive correlation with log *k*_w9.1_ suggests that more lipophilic compounds have a higher affinity for the apolar stationary phase and greater retention in HPLC.

In capillary electrophoresis at pH 9.1, the descriptors selected using the MLR method (Equation (21)) belong to the 2D matrix-based descriptors, GETAWAY, Edge adjacency indices and quantum chemical descriptors (Table 6).

SM15_EA(dm) is the spectral moment of order 15 from the edge adjacency matrix, weighted by the dipole moment, and is the only descriptor in a model that shows a positive correlation with *μ*_eff9.1_. In contrast, toSM15_EA(dm), SM1_Dz(Z) (spectral moment of order 1 from the Barysz matrix weighted by atomic number) belongs to the 2D matrix-based descriptors. ISH (standardised information content on leverage equality) is a class of GATAWEY descriptors and mainly encodes information about molecular symmetry. If all atoms have different leverage values, the molecule has no symmetry and ISH=1, otherwise a theoretically perfectly symmetric molecule has ISH=0. In the case of the tested compounds, all ISH values are above 0.82 and show a negative correlation with electrophoretic mobility, suggesting that compounds with a higher degree of symmetry have lower mobility under the given experimental conditions of background electrolyte pH 9.1. This descriptor also provides information about molecular entropy, so it can be useful in modelling physicochemical properties related to entropy and symmetry [47]. The highest occupied molecular orbital (HOMO) energy calculated using the B3LYP/3-21G(d,p) method [27] is negatively correlated with μ_eff9.1_ (Equation (21)), implying that compounds with a lower HOMO energy have greater mobility because they interact less with positively charged buffer cations against the wall of the capillary.

## Conclusions

Evaluation of the chromatographic and electrophoretic behaviour of a series of 29 imidazoline and alpha adrenergic receptors ligands at pH 4.4, 7.4, and 9.1 using QSRR and QSMR models allows several conclusions to be drawn. When comparing the statistical PLS and MLR methods used for model building, it is not possible to give preference to only one method, as the optimal models were formed by both. All selected QSRR models: PLS-QSRR (log *k*_w4.4_), PLS-QSRR (log *k*_w7.4_), MLR-QSRR (log *k*_w9.1_) and QSMR models: PLS-QSMR (*μ*_eff4.4_), MLR-QSMR (*μ*_eff7.4_), MLR-QSMR (*μ*_eff9.1_) displayed high accuracy in retention/migration prediction for internal and external test set compounds. Based on the established QSRR and QSMR models, it can be seen that different classes of descriptors appear in the models created at different pH values and for different analytical systems, such as HPLC and CE. Chemical composition of compounds, lipophilicity, and voluminosity have the strongest influence on the retention mechanism, while molecular size, mass, complexity, charge and electronic properties are valuable structural determinants for the electrophoretic mobility of the examined ligands. The created QSRR and QSMR models are a very useful predictive tools for retention and electrophoretic behaviour of new imidazoline/alpha adrenergic receptors ligands at three pH values, which could be used not only during the RP-HPLC/CE method development but also in the initial phase of design of novel imidazoline and alpha adrenergic receptors ligands with optimized physicochemical properties, such as lipophilicity (log *k*_w_ values) and charge-to-mass characteristics (μ_eff_ values).


