# Supplementary material for: Modelling of chromatographic and electrophoretic behaviour of imidazoline and alpha adrenergic receptors ligands under different acid-base conditions
Source: ADMET DMPK. 2024 May 3;12(5):737–57. doi: 10.5599/admet.2278 (PMC11542720; doi:10.5599/admet.2278)
Supplement: Supplementary file 1 [file ADMET-12-2278-S1.pdf]

ADMET & DMPK 12(5) (2024) S7-S11

Open Access : ISSN : 1848-7718

http://www.pub.iapchem.org/ojs/index.php/admet/index

Supplementary material to

Modelling of chromatographic and electrophoretic behaviour of imidazoline and alpha adrenergic receptors ligands under different acid-base conditions

Slavica Oljacic[1], Mitja Križman[2], Marija Popovic-Nikolic[1], Irena Vovk[2], Katarina Nikolic[1] and Danica Agbaba[1]

[1]*Department of Pharmaceutical Chemistry, Faculty of Pharmacy, University of Belgrade, Vojvode Stepe 450, 11000 Belgrade, Serbia*

[2]*Laboratory of Food Chemistry, National Institute of Chemistry, Hajdrihova 19, 1000 Ljubljana, Slovenia*

ADMET & DMPK **12(5)** (2024) 737-757; https://doi.org/10.5599/admet.2278

Table S1. Experimentally determined and calculated pK_a values of investigated compounds

Compound	pK_a experimental	pK_a calculated – strongest basic functional group (chem axon)	pK_a calculated - strongest acid functional group (chem axon)
Amiloride	8.72 ± 0.05 (potentiometric) [1]	7.35	11.43
Brimonidine	7.50 (potentiometric) [2]	8.32	
Carvedilol	7.97 (pH-metric) [3]	8.74	14.03
Clonidine	8.05 ± 0.05 (pH titration) [4]	8.16	
Clopamide	/	1.32	8.85
Clozapine	7.719 ± 0.015 (HPLC) [5]	7.35	15.9
Doxazosin	6.89 ± 0.57 (voltammetric) [6]	7.24	12.67
Efaroxan	10.02 (HPLC) [7]	9.06	
Ephedrine	9.65 (spectrophotometric) [8]	9.52	13.89
Guanfacine	/	8.65	11.64
Harman	7.21 (HPLC) [7]	5.97	13.72
Harmine	7.45 (spectrophotometric) [9]	6.15	13.54
Idazoxan	9.04 (HPLC) [7]	8.62	
Indapamide	8.8 ± 0.2 (potentiometric) [10]	0.097	8.85
Maprotiline	10.45 ± 0.02 (Potentiometric) [11]	10.54	
Mianserin	7.40 (Potentiometric) [12]		
Moxonidine	7.84 (HPLC) [7]	7.26	
Naphazoline	10.81 (pH spectrophotometric) [13]	10.19	
Olanzapine	6.255 ± 0.164 (HPLC) [5]	7.24	15.67
Oxymetazoline	10.62 (pH-spectrophotometric) [13]	10.15	10.91
Phenilephrine	9.17 and 10.45 (pH-spectrophotometric) [14]	9.69	9.07
Pseudoephedrine	/	9.52	13.89
Rilmenidine	/	7.11	
Tamsulosin	/	9.28	9.93
Tetrahydrozoline	10.51 ± 0.05 (pH titration) [4]	10.17	
Tizanidine	/	7.49	
Triamterene	7.16 (spectrophotometric)[15]	1.86	15.88
Tramazoline	10.66 ± 0.05 (pH titration) [4]	9.89	
Xylometazoline	10.2 ± 0.1 (pH titration) [4]	10.29	

Table S2. The obtained values of intercept (log k_w) and slope (S) at pH 4.4, 7.4 and 9.1

Compound	pH 4.4		pH 7.4		pH 9.1	
	log k_w	S	log k_w	S	log k_w	S
Amiloride	1.11 ± 0.04	-3.31 ± 0.09	1.05 ± 0.04	-3.23 ± 0.12	0.98 ± 0.04	-2.74 ± 0.11
Brimonidine	0.99 ± 0.03	-4.78 ± 0.21	1.08 ± 0.03	-3.20 ± 0.12	1.26 ± 0.07	-3.23 ± 0.19
Carvedilol	3.75 ± 0.04	-5.84 ± 0.07	4.15 ± 0.08	-5.44 ± 0.13	4.21 ± 0.10	-5.56 ± 0.18
Clonidine	0.82 ± 0.02	-2.74 ± 0.05	0.98 ± 0.01	-1.82 ± 0.02	1.47 ± 0.01	-2.71 ± 0.03
Clopamide	2.00 ± 0.02	-3.61 ± 0.04	1.99 ± 0.03	-3.59 ± 0.08	1.87 ± 0.01	-3.34 ± 0.02
Clozapine	2.94 ± 0.03	-4.69 ± 0.06	3.85 ± 0.05	-4.88 ± 0.08	3.95 ± 0.08	-5.04 ± 0.15
Doxazosin	3.42 ± 0.07	-5.56 ± 0.14	3.67 ± 0.11	-5.05 ± 0.19	3.60 ± 0.11	-4.95 ± 0.20
Efaroxan	1.31 ± 0.02	-3.20 ± 0.04	1.33± 0.03	-3.10 ± 0.14	1.85 ± 0.01	-3.38 ± 0.03
Ephedrine	0.56 ± 0.02	-2.41 ± 0.08	0.57± 0.02	-2.49 ± 0.09	1.01 ± 0.02	-2.52 ± 0.05
Guanfacine	1.73 ± 0.01	-3.47 ± 0.03	1.92 ± 0.12	-3.21 ± 0.03	2.00 ± 0.00	-3.35 ± 0.01
Harman	1.72 ± 0.06	-3.74 ± 0.18	2.76 ± 0.05	-3.70 ± 0.09	2.78 ± 0.06	-3.75 ± 0.11
Harmine	2.15 ± 0.04	-4.28 ± 0.11	2.88 ± 0.05	-3.85 ± 0.09	2.95 ± 0.07	-3.97 ± 0.12
Idazoxan	0.82 ± 0.01	-2.66 ± 0.03	0.85 ± 0.02	-1.58 ± 0.04	1.53 ± 0.01	-2.80 ± 0.01
Indapamide	2.69 ± 0.02	-4.16 ± 0.06	2.55 ± 0.02	-4.19 ± 0.04	2.35 ± 0.03	-3.78 ± 0.06
Maprotiline	2.90 ± 0.08	-4.31 ± 0.14	2.58 ± 0.12	-3.18 ± 0.20	3.09 ± 0.08	-4.20 ± 0.14
Mianserin	2.48 ± 0.03	-4.24 ± 0.07	3.91 ± 0.05	-4.82 ± 0.08	3.92 ± 0.06	-4.83 ± 0.11
Moxonidine	0.57 ± 0.03	-3.21± 0.17	0.63 ± 0.03	-2.36 ± 0.10	0.75 ± 0.04	-2.31 ± 0.10
Naphazoline	1.52 ± 0.01	-3.48 ± 0.04	1.52 ± 0.01	-3.64 ± 0.05	1.76 ± 0.01	-3.50 ± 0.03
Olanzapine	1.64 ± 0.04	-3.37 ± 0.11	3.23 ± 0.07	-4.37 ± 0.12	3.28 ± 0.08	-4.46 ± 0.14
Oxymetazoline	2.33 ± 0.02	-3.89 ± 0.04	2.33 ± 0.04	-3.61 ± 0.10	2.60 ± 0.07	-4.14 ± 0.16
Phenilephrine	0.09 ± 0.02	-2.53 ± 0.14	0.04 ± 0.01	-2.62 ± 0.06	0.39 ± 0.02	-2.00 ± 0.06
Pseudoephedrine	0.60 ± 0.03	-2.60 ± 0.10	0.61 ± 0.02	-2.67 ± 0.12	1.10 ± 0.02	-2.72 ± 0.07
Rilmenidine	0.92 ± 0.03	-2.88 ± 0.09	0.90 ± 0.03	-2.35 ± 0.11	1.48 ± 0.02	-2.96 ± 0.06
Tamsulosin	2.22 ± 0.05	-4.73 ± 0.13	2.75 ± 0.04	-4.51 ± 0.10	3.06 ± 0.09	-4.97 ± 0.20
Tetrahydrozoline	1.06 ± 0.02	-2.94 ± 0.05	1.08 ± 0.01	-3.24 ± 0.06	1.32 ± 0.03	-2.99 ± 0.07
Tizanidine	0.74 ± 0.02	-2.65 ± 0.06	1.12 ± 0.01	-2.23 ± 0.03	1.34 ± 0.01	-2.63 ± 0.04
Triamterene	1.75 ± 0.05	-3.96 ± 0.14	1.92 ± 0.05	-3.76 ± 0.12	1.93 ± 0.04	-3.75 ± 0.11
Tramazoline	1.55 ± 0.03	-3.43 ± 0.07	1.51 ± 0.01	-3.51 ± 0.06	1.74 ± 0.01	-3.37 ± 0.03
Xylometazoline	2.61 ± 0.00	-4.22 ± 0.01	2.59 ± 0.02	-4.14 ± 0.04	2.79 ± 0.08	-4.17 ± 0.16

Table S3. Values of molecular descriptors in selected QSRR models

Compound	H_Dz(p)	VE2_Dz(p)	SpMax5_Bh(e)	SpMax5_Bh(i)	SM02_EA(ri)	SM04_EA(ri)	Ho_Dt	SM3_Dt	VE2_Dt	H_D/Dt	Log *D* 7.4	nBM	SpMin6_Bh(p)	Log *D* 9.1
Amiloride	33.776	0.227	2.744	2.858	3.681	5.207	23.436	12.88	0.242	180.505	-0.44	8	0.859	-0.5
Brimonidine	55.134	0.223	2.907	2.951	3.88	5.373	34.696	14.901	0.228	406.188	-0.01	12	0.912	0.35
Carvedilol	106.5	0.168	3.462	3.514	4.416	5.919	58.541	17.873	0.168	1050.43	1.94	21	1.512	3.14
Clonidine	40.525	0.249	2.659	2.809	3.626	5.068	24.484	13.189	0.247	196.719	1.07	7	0.609	1.51
Clopamide	75.618	0.202	3.31	3.367	4.148	5.741	36.117	15.163	0.192	420.06	1.5	9	1.361	1.22
Clozapine	85.596	0.182	3.111	3.206	4.244	5.776	53.52	16.951	0.191	1003.68	3.11	13	1.263	3.38
Doxazosin	111.56	0.159	3.458	3.502	4.552	6.079	66.291	18.035	0.157	1276.76	1.63	18	1.525	2.03
Efaroxan	46.718	0.227	2.884	2.941	3.898	5.574	31.798	14.261	0.241	339.381	1.81	7	1.061	2.13
Ephedrine	30.68	0.256	2.854	2.925	3.422	4.918	19.244	11.821	0.275	116.533	-0.8	6	0.874	0.75
Guanfacine	43.851	0.232	2.821	2.868	3.666	5.165	23.447	13.019	0.244	178.329	1.5	8	0.844	1.57
Harman	54.025	0.235	2.899	2.974	3.809	5.401	33.834	14.751	0.188	438.283	2.09	15	0.873	2.1
Harmine	58.724	0.204	3.085	3.176	3.92	5.527	36.589	15.211	0.185	513.704	1.83	15	0.97	1.85
Idazoxan	36.223	0.241	2.789	2.876	3.752	5.2	31.939	14.391	0.244	342.498	0.9	7	0.879	1.05
Indapamid	92.712	0.196	3.27	3.309	4.299	5.919	43.456	16.077	0.19	610.063	1.88	15	1.219	1.47
Maprotiline	89.915	0.184	3.322	3.406	4.257	6.034	49.212	16.53	0.181	863.821	1.44	12	1.237	3
Mianserin	76.746	0.187	3.168	3.233	4.14	5.688	57.771	17.022	0.153	1133.27	3.68	12	1.34	3.98
Moxonidine	37.695	0.23	2.9	2.994	3.694	5.116	27.235	13.715	0.23	243.188	0.29	7	0.712	0.86
Naphazoline	58.845	0.229	2.87	2.947	3.86	5.364	33.312	14.678	0.238	369.54	0.27	12	0.971	1.52
Olanzapine	82.072	0.191	3.134	3.228	4.145	5.625	49.863	16.636	0.197	882.17	2.86	12	1.376	3.08
Oxymetazoline	65.537	0.2	3.091	3.165	4.086	5.834	31.198	14.279	0.209	314.9	1.52	7	1.242	2.74
Phenilephrine	28.34	0.256	2.707	2.806	3.422	4.894	19.323	11.937	0.274	120.986	-1.37	6	0.837	-0.05
Pseudoephedrine	30.68	0.256	2.854	2.925	3.422	4.918	19.244	11.821	0.275	116.533	-0.8	6	0.874	0.75
Rilmenidine	25.82	0.259	2.655	2.778	3.679	5.272	21.121	12.253	0.257	128.2	1.63	1	1.123	1.8
Tamsulosin	94.908	0.178	3.461	3.533	4.255	5.761	44.252	16.503	0.172	585.461	0.23	14	1.589	1.61
Tetrahydrozoline	48.542	0.231	2.8	2.892	3.809	5.345	31.942	14.418	0.244	351.329	0.2	7	0.963	1.47
Tizanidine	51.133	0.233	2.67	2.809	3.862	5.391	31.882	14.475	0.236	343.719	0.7	11	0.747	1.28
Triamterene	65.925	0.206	2.902	3.023	4.026	5.559	38.333	15.355	0.213	511.109	1.37	17	1.062	1.37
Tramazoline	48.982	0.232	2.994	3.094	3.819	5.276	33.312	14.678	0.238	369.54	1.23	7	1.013	1.25
Xylometazoline	62.515	0.207	3.079	3.162	4.016	5.735	29.824	14.073	0.216	282.476	1.79	7	1.239	3

Table S4. Experimentally obtained values of μ_{eff} at pH 4.4, 7.4 and 9.1

Compound	$\mu_{eff4.4}$ / 10^{-5} cm^2 V^{-1} s^{-1}	$\mu_{eff7.4}$ / 10^{-5} cm^2 V^{-1} s^{-1}	$\mu_{eff9.1}$ / 10^{-5} cm^2 V^{-1} s^{-1}
Amiloride	25.939 ± 0.080	21.786 ± 0.082	6.416 ± 0.022
Brimonidine	23.357 ± 0.043	13.247 ± 0.200	0.000 ± 0.000
Carvedilol	18.061 ± 0.029	13.057 ± 0.052	1.693 ± 0.071
Clonidine	25.952 ± 0.084	19.607 ± 0.132	2.575 ± 0.060
Clopamide	0.000 ± 0.000	0.000 ± 0.000	-9.640 ± 0.053
Clozapine	22.697 ± 0.017	9.204 ± 0.061	0.000 ± 0.000
Doxazosin	15.795 ± 0.013	3.137 ± 0.075	0.000 ± 0.000
Efaroxan	24.099 ± 0.035	22.704 ± 0.075	16.361 ± 0.178
Ephedrine	25.942 ± 0.032	23.199 ± 0.103	18.047 ± 0.070
Guanfacine	24.242± 0.002	5.083 ± 0.025	0.000 ± 0.000
Harman	28.192 ± 0.016	11.752 ± 0.034	0.000 ± 0.000
Harmine	26.410 ± 0.068	13.636 ± 0.051	1.305 ± 0.124
Idazoxan	26.969 ± 0.032	23.293 ± 0.020	6.627 ± 0.102
Indapamide	0.000 ± 0.000	0.000 ± 0.000	-10.382 ± 0.053
Maprotiline	20.559 ± 0.034	18.696 ± 0.048	19.402 ± 0.148
Mianserin	22.471 ± 0.004	12.234 ± 0.109	0.000 ± 0.000
Moxonidine	22.669 ± 0.104	10.907 ± 0.078	0.000 ± 0.000
Naphazoline	25.941 ± 0.038	24.180 ± 0.135	24.643 ± 0.049
Olanzapine	33.632 ± 0.036	10.679 ± 0.039	1.241 ± 0.029
Oxymetazoline	20.276 ± 0.040	19.061 ± 0.049	19.556 ± 0.076
Phenilephrine	25.780 ± 0.006	21.650 ±0.195	8.661 ± 0.036
Pseudoephedrine	26.633 ± 0.043	23.691 ± 0,051	9.299 ± 0.058
Rilmenidine	25.350 ± 0.121	22.551 ± 0.029	14.065 ± 0.214
Tamsulosin	17.310 ± 0.002	14.444 ± 0.058	2.472 ± 0.133
Tetrahydrozoline	25.680 ± 0.049	24.299 ± 0.057	25.047 ± 0.066
Tizanidine	24.770 ± 0.035	12.077 ± 0.042	0.000 ± 0.000
Triamterene	22.146 ± 0.019	1.140 ± 0.053	0.000 ± 0.000
Tramazoline	24.393 ± 0.067	22.988 ± 0.060	23.713 ± 0.031
Xylometazoline	21.032 ± 0.019	19.943 ± 0.006	20.627 ± 0.015

Table S5. Values of molecular descriptors in selected QSMR models

Compound	Ho_Dz(e)	ATS6s	ATS7s	ATSC6m	GGI6	R7u+	Mor28s	HATS2i	SM1_Dz(Z)	SM15_EA(dm)	HOMO B3LYP-3-21-G-d,p	ISH
Amiloride	15.452	5.096	4.849	11.518	0.203	0.024	0.25	0.216	1.064	8.807	-0.20627	0.898
Brimonidine	16.588	4.721	4.559	20.778	0.237	0.044	-0.404	0.493	0.933	3.118	-0.34699	0.971
Carvedilol	31.49	5.828	5.746	31.894	0.35	0.014	-2.082	0.316	0.827	0	-0.20165	0.845
Clonidine	14.367	4.586	4.316	11.589	0.18	0.035	-0.645	0.5	1.002	3.118	-0.3812	0.925
Clopamide	23.453	6.055	5.358	27.723	0.285	0.015	0.001	0.418	1.239	0	-0.05235	0.939
Clozapine	23.225	5.527	5.496	24.117	0.538	0.021	-1.088	0.382	0.797	2.817	-0.19694	0.885
Doxazosin	33.293	6.031	5.97	37.297	0.808	0.019	-0.941	0.332	1.087	5.589	-0.18057	0.862
Efaroxan	16.979	4.995	4.554	17.491	0.115	0.025	0.591	0.504	0.429	10.324	-0.20392	0.875
Ephedrine	13.287	4.504	3.97	9.804	0.1	0.027	0.672	0.469	0.331	0	-0.38123	0.796
Guanfacine	15.995	4.759	4.885	11.992	0.345	0.029	-0.597	0.425	1.089	4.73	-0.21344	0.932
Harman	12.859	4.267	3.714	7.381	0.08	0.051	-0.334	1.018	0.251	0	-0.21057	0.925
Harmine	15.234	4.627	4.362	10.368	0.258	0.029	-0.356	0.872	0.429	0	-0.20827	0.938
Idazoxan	14.926	4.549	4.18	10.684	0.081	0.03	0.556	0.573	0.58	9.644	-0.20796	0.885
Indapamid	24.654	6.051	5.432	22.255	0.356	0.019	0.035	0.351	1.239	0.113	-0.05221	0.909
Maprotiline	21.867	5.526	5.282	29.87	0.32	0.018	2.157	0.384	0.134	9.151	-0.30876	0.927
Mianserin	20.017	5.451	5.144	26.449	0.385	0.021	-1.364	0.385	0.251	4.933	-0.18137	0.899
Moxonidine	16.576	5.043	4.752	18.018	0.261	0.039	-1.077	0.595	0.96	6.946	-0.19384	0.938
Naphazoline	16.141	4.803	4.588	12.496	0.096	0.023	0.035	0.465	0.251	7.65	-0.33388	0.875
Olanzapine	22.532	5.423	5.347	28.823	0.498	0.018	-1.522	0.42	0.787	5.749	-0.18797	0.939
Oxymetazoline	21.65	5.603	5.341	27.125	0.354	0.017	0.912	0.396	0.429	6.194	-0.32998	0.901
Phenilephrine	13.024	4.622	4.159	9.555	0.101	0.032	-1.168	0.444	0.496	0	-0.33229	0.907
Pseudoephedrine	13.287	4.504	3.97	9.804	0.1	0.024	-0.695	0.476	0.331	0	-0.37985	0.954
Rilmenidine	14.957	4.716	4.174	14.173	0	0.02	0.418	0.601	0.429	5.595	-0.19954	0.958
Tamsulosin	30.556	5.993	5.754	42.323	0.568	0.015	0.574	0.343	1.151	0	-0.32392	0.89
Tetrahydrozoline	15.662	4.835	4.304	15.558	0.093	0.025	-0.188	0.486	0.251	9.039	-0.3598	0.829
Tizanidine	15.618	4.701	4.48	8.561	0.18	0.039	0.312	0.697	1.094	6.946	-0.07105	0.906
Triamterene	18.302	5.279	5.041	13.191	0.342	0.049	1.215	0.413	0.693	0	-0.19496	0.892
Tramazoline	16.744	4.866	4.677	14.859	0.096	0.021	-0.311	0.483	0.357	3.118	-0.35911	0.82
Xylometazoline	20.619	5.386	5.177	23.582	0.302	0.017	0.468	0.419	0.251	6.194	-0.34737	0.883

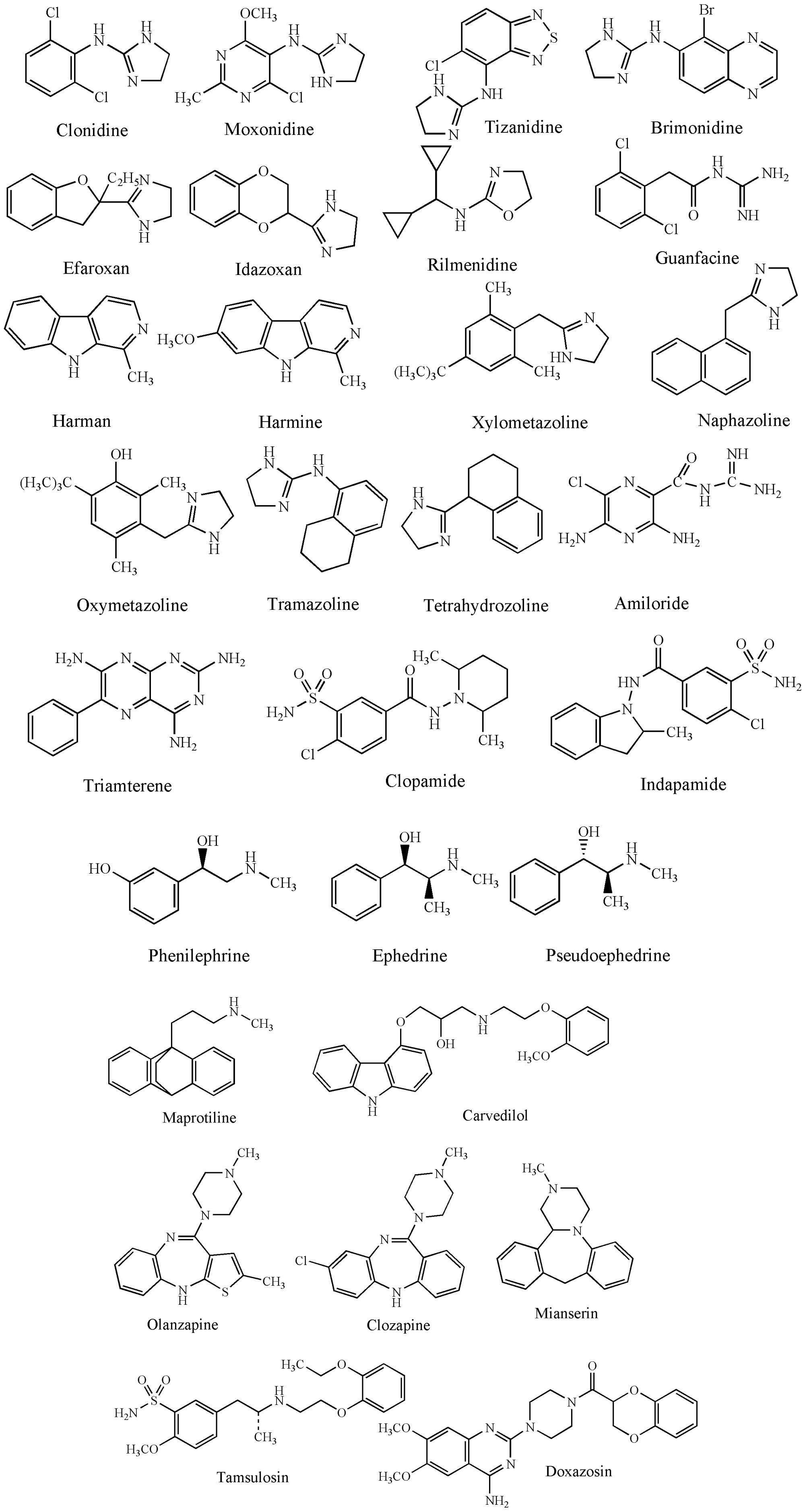

Figure S1. The chemical structures of analyzed compounds

(cc) BY

References

[1] L. Narasimham, V. D. Barhate. Physico-chemical characterization of some beta blockers and anti-diabetic drugs - potentiometric and spectrophotometric pKa determination in different co-solvents. *European Journal of Chemistry* **2** (2011) 36-46. https://doi.org/10.5155/eurjchem.2.1.36-46.371

[2] D. S. Chien, J. J. Homsy, C. Gluchowskil, D. D. S.Tang-Liu. Corneal and conjunctival/scleral penetration of p-aminoclonidine, AGN 190342, and clonidine in rabbit eyes. *Current Eye Research* **9** (1990) 1051-1059. https://doi.org/10.3109/02713689008997579

[3] G. Caron, G. Steyaert, A. Pagliara, F. Reymond, P. Crivori, P. Gaillard, P. A. Carrupt, A. Avdeef, J. Comer, K. J. Box, H. H. Girault, B. Testa. Structure-Lipophilicity Relationships of Neutral and Protonated b-Blockers Part I Intra- and Intermolecular Effects in Isotropic Solvent Systems. *Helvetica Chimica Acta* **82** (1999) 1211-1222. https://doi.org/10.1002/(SICI)1522-2675(19990804)82:8<1211::AID-HLCA1211>3.0.CO;2-K

[4] H.S. Boudier,G. Smeets, G Brouwer, J. van Rossum. Central and peripheral alpha adrenergic activity of imidazoline derivatives. *Life Sciences* **15** (1974) 887-899. https://doi.org/10.1016/0024-3205(74)90005-8

[5] S. Sanli, B. Akmese, Y. Altun. Determination of pKa values of some antipsychotic drugs by HPLC--correlations with the Kamlet and taft solvatochromic parameters and HPLC analysis in dosage forms. *Journal of AOAC International* **96** (2013) 60-66. https://doi.org/10.5740/jaoacint.11-494.

[6] A. Arra, S. Fernández de Betoño, J. M. Moreda, A. Cid, J. F. Arranz. Cathodic stripping voltammetric determination of doxazosin in urine and pharmaceutical tablets using carbon paste electrodes. *Analyst* **122** (1997) 849-854. https://doi.org/10.1039/a701210a.

[7] P. Wiczling, A. Nasal, Ł. Kubik, R. Kaliszan. A new pH/organic modifier gradient RP HPLC method for convenient determination of lipophilicity and acidity of drugs as applied to established imidazoline agents. *European Journal of Pharmaceutical Sciences* **47** (2012) 1-5. https://doi.org/10.1016/j.ejps.2012.04.021

[8] K.Y. Tama, K. Takács-Novák. Multi-wavelength spectrophotometric determination of acid dissociation constants: a validation study. *Analytica Chimica Acta* **434** (2001) 157-167. https://doi.org/10.1016/S0003-2670(01)00810-8

[9] T. Douglas, R. K. Sharma, J. F. Walmsley, R. C.Hider. Ionization processes of some harmala alkaloids. *Molecular Pharmacology* **23** (1983) 614-618.

[10] T. J. DiFeo, J. E. Shuster. Indapamide. *Analytical Profiles of Drug Substancesa and Excipients* **23** (1994) 229-268. https://doi.org/10.1016/S0099-5428(08)60604-8

[11] R. Ruiz, C. Ràfols, M. Rosés, E. Bosch. A potentially simpler approach to measure aqueous pKa of insoluble basic drugs containing amino groups. *Journal of Pharmaceutical Sciences* **92** (2003) 1473-1481. doi: 10.1002/jps.10415.

[12] J. Kelder, C. Funke, T. De Boer, L. Delbressine, D. Leysen, V. Nickolson. A comparison of the physicochemical and biological properties of mirtazapine and mianserin. *Journal of Pharmacy and Pharmacology* **49** (1997) 403-411. https://doi.org/10.1111/j.2042-7158.1997.tb06814.x

[13] M. Meloun, T. Syrový, A. Vrána. The thermodynamic dissociation constants of ambroxol, antazoline, naphazoline, oxymetazoline and ranitidine by the regression analysis of spectrophotometric data. *Talanta* **62** (2004) 511-522. https://doi.org/10.1016/j.talanta.2003.08.027

[14] M. Meloun, T. Syrový, A. Vrána. The thermodynamic dissociation constants of losartan, paracetamol, phenylephrine and quinine by the regression analysis of spectrophotometric data. *Analytica Chimica Acta* **533** (2005) 97-110. https://doi.org/10.1016/j.aca.2004.11.007

[15] U. Domańska, A. Pobudkowska, A Pelczarska, L. Zukowski. Modelling, solubility and pK(a) of five sparingly soluble drugs. *International Journal of Pharmaceutics* **403** (2011) 115-122. https://doi.org/10.1016/j.ijpharm.2010.10.034
