# Supplementary material for: Modelling of chromatographic and electrophoretic behaviour of imidazoline and alpha adrenergic receptors ligands under different acid-base conditions
Source: ADMET DMPK. 2024 May 3;12(5):737–57. doi: 10.5599/admet.2278 (PMC11542720; doi:10.5599/admet.2278)
Supplement: Supplementary file 2 [file ADMET-12-2278-S1.docx]

*ADMET & DMPK 12(5) (2024) S7-S11*

*
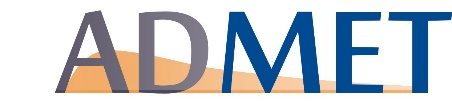
***Open Access : ISSN : 1848-7718**[***http://www.pub.iapchem.org/ojs/index.php/admet/index***](http://www.pub.iapchem.org/ojs/index.php/admet/index)

Supplementary material to

Modelling of chromatographic and electrophoretic behaviour of imidazoline and alpha adrenergic receptors ligands under different acid-base conditions

Slavica Oljacic^1^, Mitja Križman^2^, Marija Popovic-Nikolic^1^, Irena Vovk^2^,
Katarina Nikolic^1^ and Danica Agbaba^1^

^1^Department of Pharmaceutical Chemistry, Faculty of Pharmacy, University of Belgrade, Vojvode Stepe 450,
11000 Belgrade, Serbia
^2^Laboratory of Food Chemistry, National Institute of Chemistry, Hajdrihova 19, 1000 Ljubljana, Slovenia

ADMET & DMPK **12(5)** (2024) 737-757; <https://doi.org/10.5599/admet.2278>

**Table S1.** Experimentally determined and calculated p*K*_a_ values of investigated compounds

| Compound | p*K*_a_ experimental | p*K*_a_ calculated – strongest basic functional group (chem axon) | p*K*_a_ calculated - strongest acid functional group (chem axon) |
| --- | --- | --- | --- |
| Amiloride | 8.72 ± 0.05 (potentiometric) [1] | 7.35 | 11.43 |
| Brimonidine | 7.50 (potentiometric) [2] | 8.32 |  |
| Carvedilol | 7.97 (pH-metric) [3] | 8.74 | 14.03 |
| Clonidine | 8.05 ± 0.05 (pH titration) [4] | 8.16 |  |
| Clopamide | / | 1.32 | 8.85 |
| Clozapine | 7.719 ± 0.015 (HPLC) [5] | 7.35 | 15.9 |
| Doxazosin | 6.89 ± 0.57 (voltammetric) [6] | 7.24 | 12.67 |
| Efaroxan | 10.02 (HPLC) [7] | 9.06 |  |
| Ephedrine | 9.65 (spectrophotometric) [8] | 9.52 | 13.89 |
| Guanfacine | / | 8.65 | 11.64 |
| Harman | 7.21 (HPLC) [7] | 5.97 | 13.72 |
| Harmine | 7.45 (spectrophotometric) [9] | 6.15 | 13.54 |
| Idazoxan | 9.04 (HPLC) [7] | 8.62 |  |
| Indapamide | 8.8 ± 0.2 (potentiometric) [10] | 0.097 | 8.85 |
| Maprotiline | 10.45 ± 0.02 (Potentiometric) [11] | 10.54 |  |
| Mianserin | 7.40 (Potentiometric) [12] |  |  |
| Moxonidine | 7.84 (HPLC) [7] | 7.26 |  |
| Naphazoline | 10.81 (pH spectrophotometric) [13 ] | 10.19 |  |
| Olanzapine | 6.255 ± 0.164 (HPLC) [5] | 7.24 | 15.67 |
| Oxymetazoline | 10.62 (pH-spectrophotometric) [13] | 10.15 | 10.91 |
| Phenilephrine | 9.17 and 10.45 (pH-spectrophotometric) [14 ] | 9.69 | 9.07 |
| Pseudoephedrine | / | 9.52 | 13.89 |
| Rilmenidine | / | 7.11 |  |
| Tamsulosin | / | 9.28 | 9.93 |
| Tetrahydrozoline | 10.51 ± 0.05 (pH titration) [4] | 10.17 |  |
| Tizanidine | / | 7.49 |  |
| Triamterene | 7.16 (spectrophotometric )[15] | 1.86 | 15.88 |
| Tramazoline | 10.66 ± 0.05 (pH titration) [4] | 9.89 |  |
| Xylometazoline | 10.2 ± 0.1 (pH titration) [4] | 10.29 |  |

**Table S2.** The obtained values of intercept (log *k*_w_) and slope (*S*) at pH 4.4, 7.4 and 9.1

| Compound | pH 4.4 | | pH 7.4 | | pH 9.1 | |
| --- | --- | --- | --- | --- | --- | --- |
|  | log *k*_w_ | *S* | log *k*_w_ | *S* | log *k*_w_ | *S* |
| Amiloride | 1.11 ± 0.04 | -3.31 ± 0.09 | 1.05 ± 0.04 | -3.23 ± 0.12 | 0.98 ± 0.04 | -2.74 ± 0.11 |
| Brimonidine | 0.99 ± 0.03 | -4.78 ± 0.21 | 1.08 ± 0.03 | -3.20 ± 0.12 | 1.26 ± 0.07 | -3.23 ± 0.19 |
| Carvedilol | 3.75 ± 0.04 | -5.84 ± 0.07 | 4.15 ± 0.08 | -5.44 ± 0.13 | 4.21 ± 0.10 | -5.56 ± 0.18 |
| Clonidine | 0.82 ± 0.02 | -2.74 ± 0.05 | 0.98 ± 0.01 | -1.82 ± 0.02 | 1.47 ± 0.01 | -2.71 ± 0.03 |
| Clopamide | 2.00 ± 0.02 | -3.61 ± 0.04 | 1.99 ± 0.03 | -3.59 ± 0.08 | 1.87 ± 0.01 | -3.34 ± 0.02 |
| Clozapine | 2.94 ± 0.03 | -4.69 ± 0.06 | 3.85 ± 0.05 | -4.88 ± 0.08 | 3.95 ± 0.08 | -5.04 ± 0.15 |
| Doxazosin | 3.42 ± 0.07 | -5.56 ± 0.14 | 3.67 ± 0.11 | -5.05 ± 0.19 | 3.60 ± 0.11 | -4.95 ± 0.20 |
| Efaroxan | 1.31 ± 0.02 | -3.20 ± 0.04 | 1.33± 0.03 | -3.10 ± 0.14 | 1.85 ± 0.01 | -3.38 ± 0.03 |
| Ephedrine | 0.56 ± 0.02 | -2.41 ± 0.08 | 0.57± 0.02 | -2.49 ± 0.09 | 1.01 ± 0.02 | -2.52 ± 0.05 |
| Guanfacine | 1.73 ± 0.01 | -3.47 ± 0.03 | 1.92 ± 0.12 | -3.21 ± 0.03 | 2.00 ± 0.00 | -3.35 ± 0.01 |
| Harman | 1.72 ± 0.06 | -3.74 ± 0.18 | 2.76 ± 0.05 | -3.70 ± 0.09 | 2.78 ± 0.06 | -3.75 ± 0.11 |
| Harmine | 2.15 ± 0.04 | -4.28 ± 0.11 | 2.88 ± 0.05 | -3.85 ± 0.09 | 2.95 ± 0.07 | -3.97 ± 0.12 |
| Idazoxan | 0.82 ± 0.01 | -2.66 ± 0.03 | 0.85 ± 0.02 | -1.58 ± 0.04 | 1.53 ± 0.01 | -2.80 ± 0.01 |
| Indapamide | 2.69 ± 0.02 | -4.16 ± 0.06 | 2.55 ± 0.02 | -4.19 ± 0.04 | 2.35 ± 0.03 | -3.78 ± 0.06 |
| Maprotiline | 2.90 ± 0.08 | -4.31 ± 0.14 | 2.58 ± 0.12 | -3.18 ± 0.20 | 3.09 ± 0.08 | -4.20 ± 0.14 |
| Mianserin | 2.48 ± 0.03 | -4.24 ± 0.07 | 3.91 ± 0.05 | -4.82 ± 0.08 | 3.92 ± 0.06 | -4.83 ± 0.11 |
| Moxonidine | 0.57 ± 0.03 | -3.21± 0.17 | 0.63 ± 0.03 | -2.36 ± 0.10 | 0.75 ± 0.04 | -2.31 ± 0.10 |
| Naphazoline | 1.52 ± 0.01 | -3.48 ± 0.04 | 1.52 ± 0.01 | -3.64 ± 0.05 | 1.76 ± 0.01 | -3.50 ± 0.03 |
| Olanzapine | 1.64 ± 0.04 | -3.37 ± 0.11 | 3.23 ± 0.07 | -4.37 ± 0.12 | 3.28 ± 0.08 | -4.46 ± 0.14 |
| Oxymetazoline | 2.33 ± 0.02 | -3.89 ± 0.04 | 2.33 ± 0.04 | -3.61 ± 0.10 | 2.60 ± 0.07 | -4.14 ± 0.16 |
| Phenilephrine | 0.09 ± 0.02 | -2.53 ± 0.14 | 0.04 ± 0.01 | -2.62 ± 0.06 | 0.39 ± 0.02 | -2.00 ± 0.06 |
| Pseudoephedrine | 0.60 ± 0.03 | -2.60 ± 0.10 | 0.61 ± 0.02 | -2.67 ± 0.12 | 1.10 ± 0.02 | -2.72 ± 0.07 |
| Rilmenidine | 0.92 ± 0.03 | -2.88 ± 0.09 | 0.90 ± 0.03 | -2.35 ± 0.11 | 1.48 ± 0.02 | -2.96 ± 0.06 |
| Tamsulosin | 2.22 ± 0.05 | -4.73 ± 0.13 | 2.75 ± 0.04 | -4.51 ± 0.10 | 3.06 ± 0.09 | -4.97 ± 0.20 |
| Tetrahydrozoline | 1.06 ± 0.02 | -2.94 ± 0.05 | 1.08 ± 0.01 | -3.24 ± 0.06 | 1.32 ± 0.03 | -2.99 ± 0.07 |
| Tizanidine | 0.74 ± 0.02 | -2.65 ± 0.06 | 1.12 ± 0.01 | -2.23 ± 0.03 | 1.34 ± 0.01 | -2.63 ± 0.04 |
| Triamterene | 1.75 ± 0.05 | -3.96 ± 0.14 | 1.92 ± 0.05 | -3.76 ± 0.12 | 1.93 ± 0.04 | -3.75 ± 0.11 |
| Tramazoline | 1.55 ± 0.03 | -3.43 ± 0.07 | 1.51 ± 0.01 | -3.51 ± 0.06 | 1.74 ± 0.01 | -3.37 ± 0.03 |
| Xylometazoline | 2.61 ± 0.00 | -4.22 ± 0.01 | 2.59 ± 0.02 | -4.14 ± 0.04 | 2.79 ± 0.08 | -4.17 ± 0.16 |

**Table S3.** Values of molecular descriptors in selected QSRR models

| Compound | H_Dz(p) | VE2_Dz(p) | SpMax5_Bh(e) | SpMax5_Bh(i) | SM02_EA(ri) | SM04_EA(ri) | Ho_Dt | SM3_Dt | VE2_Dt | H_D/Dt | Log *D* 7.4 | nBM | SpMin6_Bh(p) | Log *D* 9.1 |
| --- | --- | --- | --- | --- | --- | --- | --- | --- | --- | --- | --- | --- | --- | --- |
| Amiloride | 33.776 | 0.227 | 2.744 | 2.858 | 3.681 | 5.207 | 23.436 | 12.88 | 0.242 | 180.505 | -0.44 | 8 | 0.859 | -0.5 |
| Brimonidine | 55.134 | 0.223 | 2.907 | 2.951 | 3.88 | 5.373 | 34.696 | 14.901 | 0.228 | 406.188 | -0.01 | 12 | 0.912 | 0.35 |
| Carvedilol | 106.5 | 0.168 | 3.462 | 3.514 | 4.416 | 5.919 | 58.541 | 17.873 | 0.168 | 1050.43 | 1.94 | 21 | 1.512 | 3.14 |
| Clonidine | 40.525 | 0.249 | 2.659 | 2.809 | 3.626 | 5.068 | 24.484 | 13.189 | 0.247 | 196.719 | 1.07 | 7 | 0.609 | 1.51 |
| Clopamide | 75.618 | 0.202 | 3.31 | 3.367 | 4.148 | 5.741 | 36.117 | 15.163 | 0.192 | 420.06 | 1.5 | 9 | 1.361 | 1.22 |
| Clozapine | 85.596 | 0.182 | 3.111 | 3.206 | 4.244 | 5.776 | 53.52 | 16.951 | 0.191 | 1003.68 | 3.11 | 13 | 1.263 | 3.38 |
| Doxazosin | 111.56 | 0.159 | 3.458 | 3.502 | 4.552 | 6.079 | 66.291 | 18.035 | 0.157 | 1276.76 | 1.63 | 18 | 1.525 | 2.03 |
| Efaroxan | 46.718 | 0.227 | 2.884 | 2.941 | 3.898 | 5.574 | 31.798 | 14.261 | 0.241 | 339.381 | 1.81 | 7 | 1.061 | 2.13 |
| Ephedrine | 30.68 | 0.256 | 2.854 | 2.925 | 3.422 | 4.918 | 19.244 | 11.821 | 0.275 | 116.533 | -0.8 | 6 | 0.874 | 0.75 |
| Guanfacine | 43.851 | 0.232 | 2.821 | 2.868 | 3.666 | 5.165 | 23.447 | 13.019 | 0.244 | 178.329 | 1.5 | 8 | 0.844 | 1.57 |
| Harman | 54.025 | 0.235 | 2.899 | 2.974 | 3.809 | 5.401 | 33.834 | 14.751 | 0.188 | 438.283 | 2.09 | 15 | 0.873 | 2.1 |
| Harmine | 58.724 | 0.204 | 3.085 | 3.176 | 3.92 | 5.527 | 36.589 | 15.211 | 0.185 | 513.704 | 1.83 | 15 | 0.97 | 1.85 |
| Idazoxan | 36.223 | 0.241 | 2.789 | 2.876 | 3.752 | 5.2 | 31.939 | 14.391 | 0.244 | 342.498 | 0.9 | 7 | 0.879 | 1.05 |
| Indapamid | 92.712 | 0.196 | 3.27 | 3.309 | 4.299 | 5.919 | 43.456 | 16.077 | 0.19 | 610.063 | 1.88 | 15 | 1.219 | 1.47 |
| Maprotiline | 89.915 | 0.184 | 3.322 | 3.406 | 4.257 | 6.034 | 49.212 | 16.53 | 0.181 | 863.821 | 1.44 | 12 | 1.237 | 3 |
| Mianserin | 76.746 | 0.187 | 3.168 | 3.233 | 4.14 | 5.688 | 57.771 | 17.022 | 0.153 | 1133.27 | 3.68 | 12 | 1.34 | 3.98 |
| Moxonidine | 37.695 | 0.23 | 2.9 | 2.994 | 3.694 | 5.116 | 27.235 | 13.715 | 0.23 | 243.188 | 0.29 | 7 | 0.712 | 0.86 |
| Naphazoline | 58.845 | 0.229 | 2.87 | 2.947 | 3.86 | 5.364 | 33.312 | 14.678 | 0.238 | 369.54 | 0.27 | 12 | 0.971 | 1.52 |
| Olanzapine | 82.072 | 0.191 | 3.134 | 3.228 | 4.145 | 5.625 | 49.863 | 16.636 | 0.197 | 882.17 | 2.86 | 12 | 1.376 | 3.08 |
| Oxymetazoline | 65.537 | 0.2 | 3.091 | 3.165 | 4.086 | 5.834 | 31.198 | 14.279 | 0.209 | 314.9 | 1.52 | 7 | 1.242 | 2.74 |
| Phenilephrine | 28.34 | 0.256 | 2.707 | 2.806 | 3.422 | 4.894 | 19.323 | 11.937 | 0.274 | 120.986 | -1.37 | 6 | 0.837 | -0.05 |
| Pseudoephedrine | 30.68 | 0.256 | 2.854 | 2.925 | 3.422 | 4.918 | 19.244 | 11.821 | 0.275 | 116.533 | -0.8 | 6 | 0.874 | 0.75 |
| Rilmenidine | 25.82 | 0.259 | 2.655 | 2.778 | 3.679 | 5.272 | 21.121 | 12.253 | 0.257 | 128.2 | 1.63 | 1 | 1.123 | 1.8 |
| Tamsulosin | 94.908 | 0.178 | 3.461 | 3.533 | 4.255 | 5.761 | 44.252 | 16.503 | 0.172 | 585.461 | 0.23 | 14 | 1.589 | 1.61 |
| Tetrahydrozoline | 48.542 | 0.231 | 2.8 | 2.892 | 3.809 | 5.345 | 31.942 | 14.418 | 0.244 | 351.329 | 0.2 | 7 | 0.963 | 1.47 |
| Tizanidine | 51.133 | 0.233 | 2.67 | 2.809 | 3.862 | 5.391 | 31.882 | 14.475 | 0.236 | 343.719 | 0.7 | 11 | 0.747 | 1.28 |
| Triamterene | 65.925 | 0.206 | 2.902 | 3.023 | 4.026 | 5.559 | 38.333 | 15.355 | 0.213 | 511.109 | 1.37 | 17 | 1.062 | 1.37 |
| Tramazoline | 48.982 | 0.232 | 2.994 | 3.094 | 3.819 | 5.276 | 33.312 | 14.678 | 0.238 | 369.54 | 1.23 | 7 | 1.013 | 1.25 |
| Xylometazoline | 62.515 | 0.207 | 3.079 | 3.162 | 4.016 | 5.735 | 29.824 | 14.073 | 0.216 | 282.476 | 1.79 | 7 | 1.239 | 3 |

**Table S4.** Experimentally obtained values of *µ*_eff_ at pH 4.4, 7.4 and 9.1

| Compound | *µ*_eff4.4_ / 10^-5^ cm^2^ V^-1^ s^-1^ | *µ*_eff7.4_ / 10^-5^ cm^2^ V^-1^ s^-1^ | *µ*_eff9.1_ / 10^-5^ cm^2^ V^-1^ s^-1^ |
| --- | --- | --- | --- |
| Amiloride | 25.939 ± 0.080 | 21.786 ± 0.082 | 6.416 ± 0.022 |
| Brimonidine | 23.357 ± 0.043 | 13.247 ± 0.200 | 0.000 ± 0.000 |
| Carvedilol | 18.061 ± 0.029 | 13.057 ± 0.052 | 1.693 ± 0.071 |
| Clonidine | 25.952 ± 0.084 | 19.607 ± 0.132 | 2.575 ± 0.060 |
| Clopamide | 0.000 ± 0.000 | 0.000 ± 0.000 | -9.640 ± 0.053 |
| Clozapine | 22.697 ± 0.017 | 9.204 ± 0.061 | 0.000 ± 0.000 |
| Doxazosin | 15.795 ± 0.013 | 3.137 ± 0.075 | 0.000 ± 0.000 |
| Efaroxan | 24.099 ± 0.035 | 22.704 ± 0.075 | 16.361 ± 0.178 |
| Ephedrine | 25.942 ± 0.032 | 23.199 ± 0.103 | 18.047 ± 0.070 |
| Guanfacine | 24.242± 0.002 | 5.083 ± 0.025 | 0.000 ± 0.000 |
| Harman | 28.192 ± 0.016 | 11.752 ± 0.034 | 0.000 ± 0.000 |
| Harmine | 26.410 ± 0.068 | 13.636 ± 0.051 | 1.305 ± 0.124 |
| Idazoxan | 26.969 ± 0.032 | 23.293 ± 0.020 | 6.627 ± 0.102 |
| Indapamide | 0.000 ± 0.000 | 0.000 ± 0.000 | -10.382 ± 0.053 |
| Maprotiline | 20.559 ± 0.034 | 18.696 ± 0.048 | 19.402 ± 0.148 |
| Mianserin | 22.471 ± 0.004 | 12.234 ± 0.109 | 0.000 ± 0.000 |
| Moxonidine | 22.669 ± 0.104 | 10.907 ± 0.078 | 0.000 ± 0.000 |
| Naphazoline | 25.941 ± 0.038 | 24.180 ± 0.135 | 24.643 ± 0.049 |
| Olanzapine | 33.632 ± 0.036 | 10.679 ± 0.039 | 1.241 ± 0.029 |
| Oxymetazoline | 20.276 ± 0.040 | 19.061 ± 0.049 | 19.556 ± 0.076 |
| Phenilephrine | 25.780 ± 0.006 | 21.650 ±0.195 | 8.661 ± 0.036 |
| Pseudoephedrine | 26.633 ± 0.043 | 23.691 ± 0,051 | 9.299 ± 0.058 |
| Rilmenidine | 25.350 ± 0.121 | 22.551 ± 0.029 | 14.065 ± 0.214 |
| Tamsulosin | 17.310 ± 0.002 | 14.444 ± 0.058 | 2.472 ± 0.133 |
| Tetrahydrozoline | 25.680 ± 0.049 | 24.299 ± 0.057 | 25.047 ± 0.066 |
| Tizanidine | 24.770 ± 0.035 | 12.077 ± 0.042 | 0.000 ± 0.000 |
| Triamterene | 22.146 ± 0.019 | 1.140 ± 0.053 | 0.000 ± 0.000 |
| Tramazoline | 24.393 ± 0.067 | 22.988 ± 0.060 | 23.713 ± 0.031 |
| Xylometazoline | 21.032 ± 0.019 | 19.943 ± 0.006 | 20.627 ± 0.015 |

**Table S5.** Values of molecular descriptors in selected QSMR models

| Compound | Ho_Dz(e) | ATS6s | ATS7s | ATSC6m | GGI6 | R7u+ | Mor28s | HATS2i | SM1_Dz(Z) | SM15_EA(dm) | HOMO B3LYP- -3-21-G-d,p | ISH |
| --- | --- | --- | --- | --- | --- | --- | --- | --- | --- | --- | --- | --- |
| Amiloride | 15.452 | 5.096 | 4.849 | 11.518 | 0.203 | 0.024 | 0.25 | 0.216 | 1.064 | 8.807 | -0.20627 | 0.898 |
| Brimonidine | 16.588 | 4.721 | 4.559 | 20.778 | 0.237 | 0.044 | -0.404 | 0.493 | 0.933 | 3.118 | -0.34699 | 0.971 |
| Carvedilol | 31.49 | 5.828 | 5.746 | 31.894 | 0.35 | 0.014 | -2.082 | 0.316 | 0.827 | 0 | -0.20165 | 0.845 |
| Clonidine | 14.367 | 4.586 | 4.316 | 11.589 | 0.18 | 0.035 | -0.645 | 0.5 | 1.002 | 3.118 | -0.3812 | 0.925 |
| Clopamide | 23.453 | 6.055 | 5.358 | 27.723 | 0.285 | 0.015 | 0.001 | 0.418 | 1.239 | 0 | -0.05235 | 0.939 |
| Clozapine | 23.225 | 5.527 | 5.496 | 24.117 | 0.538 | 0.021 | -1.088 | 0.382 | 0.797 | 2.817 | -0.19694 | 0.885 |
| Doxazosin | 33.293 | 6.031 | 5.97 | 37.297 | 0.808 | 0.019 | -0.941 | 0.332 | 1.087 | 5.589 | -0.18057 | 0.862 |
| Efaroxan | 16.979 | 4.995 | 4.554 | 17.491 | 0.115 | 0.025 | 0.591 | 0.504 | 0.429 | 10.324 | -0.20392 | 0.875 |
| Ephedrine | 13.287 | 4.504 | 3.97 | 9.804 | 0.1 | 0.027 | 0.672 | 0.469 | 0.331 | 0 | -0.38123 | 0.796 |
| Guanfacine | 15.995 | 4.759 | 4.885 | 11.992 | 0.345 | 0.029 | -0.597 | 0.425 | 1.089 | 4.73 | -0.21344 | 0.932 |
| Harman | 12.859 | 4.267 | 3.714 | 7.381 | 0.08 | 0.051 | -0.334 | 1.018 | 0.251 | 0 | -0.21057 | 0.925 |
| Harmine | 15.234 | 4.627 | 4.362 | 10.368 | 0.258 | 0.029 | -0.356 | 0.872 | 0.429 | 0 | -0.20827 | 0.938 |
| Idazoxan | 14.926 | 4.549 | 4.18 | 10.684 | 0.081 | 0.03 | 0.556 | 0.573 | 0.58 | 9.644 | -0.20796 | 0.885 |
| Indapamid | 24.654 | 6.051 | 5.432 | 22.255 | 0.356 | 0.019 | 0.035 | 0.351 | 1.239 | 0.113 | -0.05221 | 0.909 |
| Maprotiline | 21.867 | 5.526 | 5.282 | 29.87 | 0.32 | 0.018 | 2.157 | 0.384 | 0.134 | 9.151 | -0.30876 | 0.927 |
| Mianserin | 20.017 | 5.451 | 5.144 | 26.449 | 0.385 | 0.021 | -1.364 | 0.385 | 0.251 | 4.933 | -0.18137 | 0.899 |
| Moxonidine | 16.576 | 5.043 | 4.752 | 18.018 | 0.261 | 0.039 | -1.077 | 0.595 | 0.96 | 6.946 | -0.19384 | 0.938 |
| Naphazoline | 16.141 | 4.803 | 4.588 | 12.496 | 0.096 | 0.023 | 0.035 | 0.465 | 0.251 | 7.65 | -0.33388 | 0.875 |
| Olanzapine | 22.532 | 5.423 | 5.347 | 28.823 | 0.498 | 0.018 | -1.522 | 0.42 | 0.787 | 5.749 | -0.18797 | 0.939 |
| Oxymetazoline | 21.65 | 5.603 | 5.341 | 27.125 | 0.354 | 0.017 | 0.912 | 0.396 | 0.429 | 6.194 | -0.32998 | 0.901 |
| Phenilephrine | 13.024 | 4.622 | 4.159 | 9.555 | 0.101 | 0.032 | -1.168 | 0.444 | 0.496 | 0 | -0.33229 | 0.907 |
| Pseudoephedrine | 13.287 | 4.504 | 3.97 | 9.804 | 0.1 | 0.024 | -0.695 | 0.476 | 0.331 | 0 | -0.37985 | 0.954 |
| Rilmenidine | 14.957 | 4.716 | 4.174 | 14.173 | 0 | 0.02 | 0.418 | 0.601 | 0.429 | 5.595 | -0.19954 | 0.958 |
| Tamsulosin | 30.556 | 5.993 | 5.754 | 42.323 | 0.568 | 0.015 | 0.574 | 0.343 | 1.151 | 0 | -0.32392 | 0.89 |
| Tetrahydrozoline | 15.662 | 4.835 | 4.304 | 15.558 | 0.093 | 0.025 | -0.188 | 0.486 | 0.251 | 9.039 | -0.3598 | 0.829 |
| Tizanidine | 15.618 | 4.701 | 4.48 | 8.561 | 0.18 | 0.039 | 0.312 | 0.697 | 1.094 | 6.946 | -0.07105 | 0.906 |
| Triamterene | 18.302 | 5.279 | 5.041 | 13.191 | 0.342 | 0.049 | 1.215 | 0.413 | 0.693 | 0 | -0.19496 | 0.892 |
| Tramazoline | 16.744 | 4.866 | 4.677 | 14.859 | 0.096 | 0.021 | -0.311 | 0.483 | 0.357 | 3.118 | -0.35911 | 0.82 |
| Xylometazoline | 20.619 | 5.386 | 5.177 | 23.582 | 0.302 | 0.017 | 0.468 | 0.419 | 0.251 | 6.194 | -0.34737 | 0.883 |


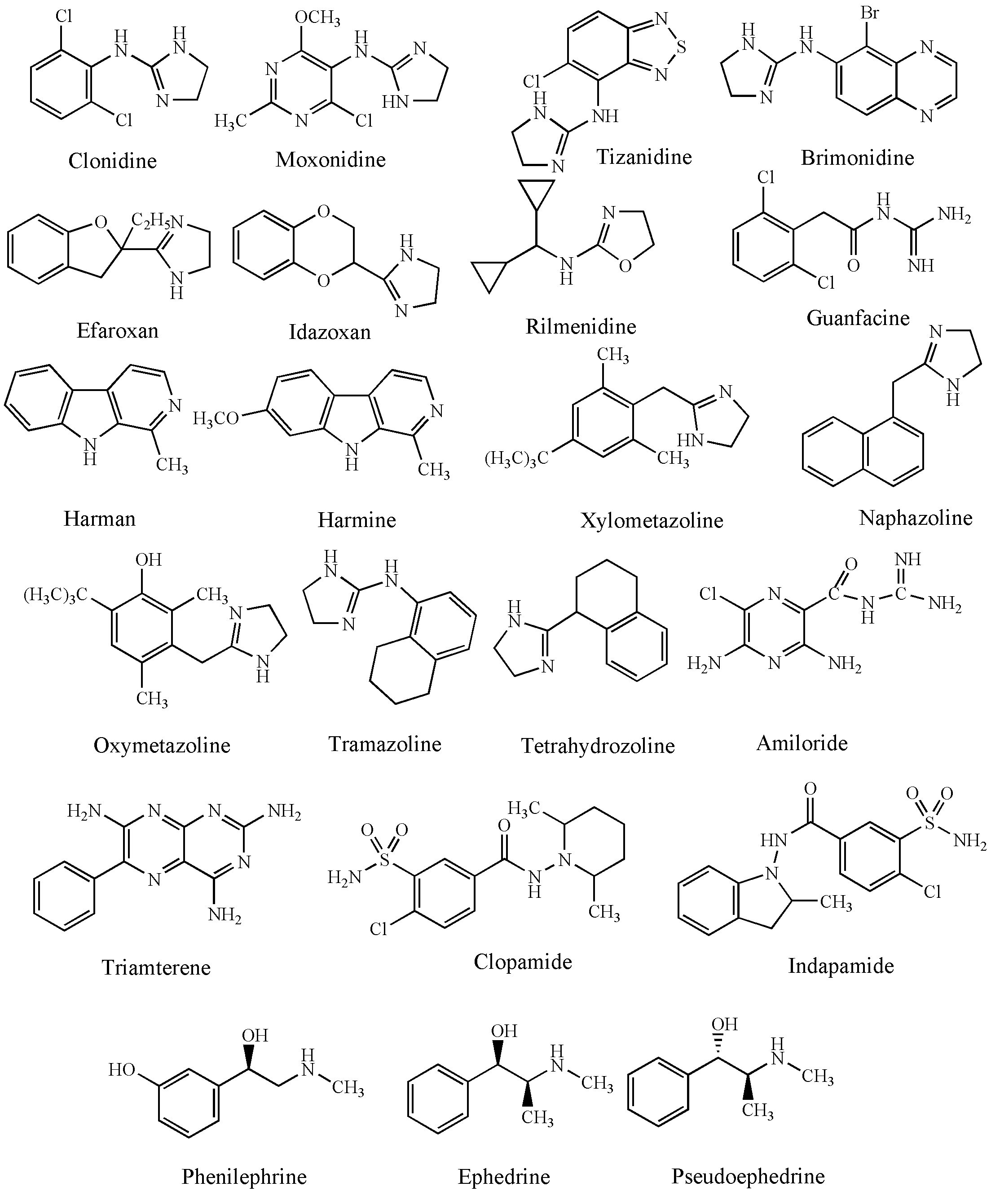

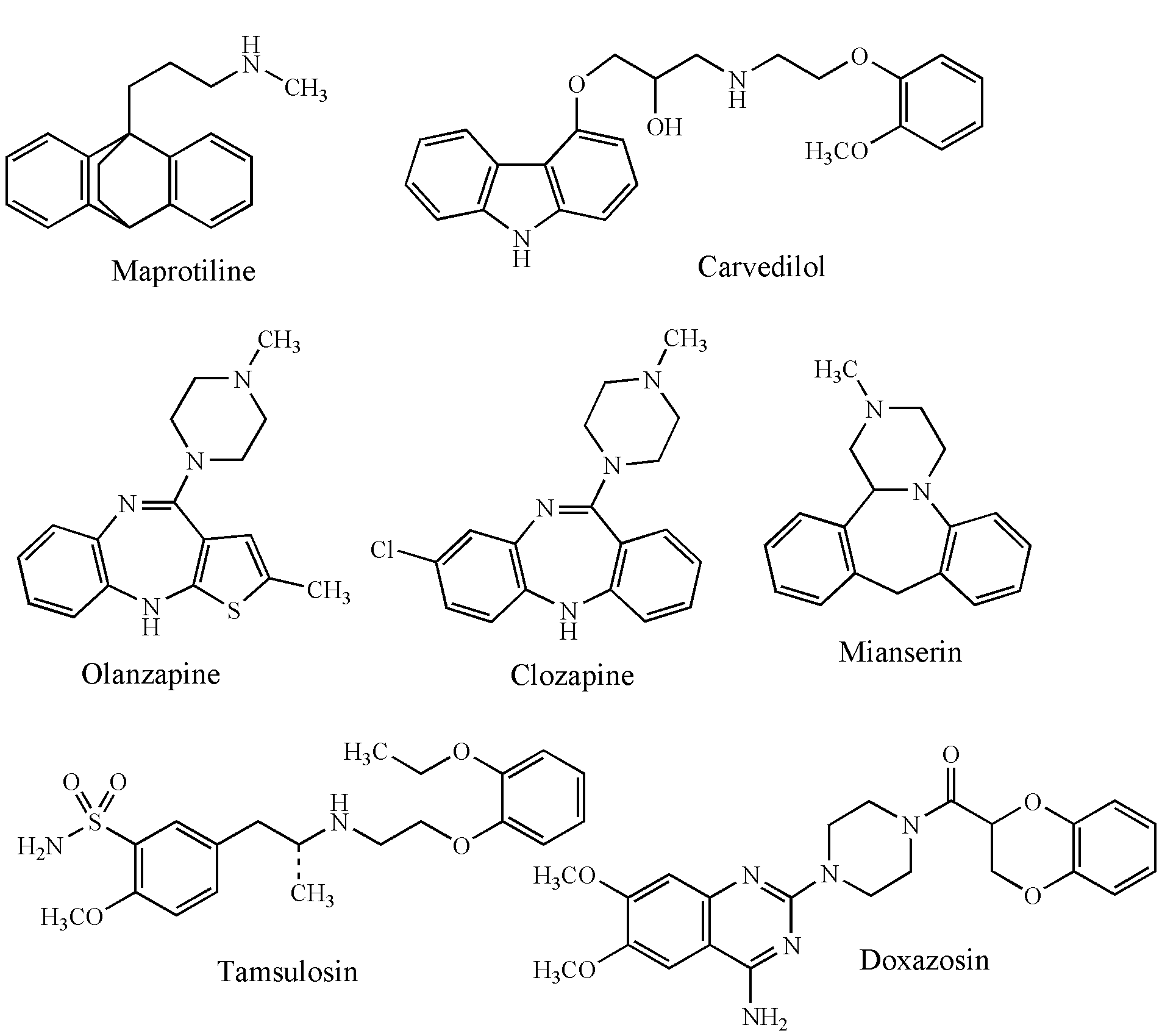


**Figure S1**. The chemical structures of analyzed compounds

References

1. L. Narasimham, V. D. Barhate. Physico‐chemical characterization of some beta blockers and anti‐diabetic drugs ‐ potentiometric and spectrophotometric pKa determination in different co‐solvents. *European Journal of Chemistry* **2** (2011) 36‐46. <https://doi.org/10.5155/eurjchem.2.1.36‐46.371>
2. D. S. Chien, J. J. Homsy, C. Gluchowskil, D. D. S.Tang-Liu. Corneal and conjunctival/scleral penetration of p-aminoclonidine, AGN 190342, and clonidine in rabbit eyes. *Current Eye Research* **9** (1990) 1051-1059. <https://doi.org/10.3109/02713689008997579>
3. G. Caron, G. Steyaert, A. Pagliara, F. Reymond, P. Crivori, P. Gaillard, P. A. Carrupt, A. Avdeef, J. Comer, K. J. Box, H. H. Girault, B. Testa. Structure-Lipophilicity Relationships of Neutral and Protonated b-Blockers Part I Intra- and Intermolecular Effects in Isotropic Solvent Systems. *Helvetica Chimica Acta* **82** (1999) 1211-1222. [https://doi.org/10.1002/(SICI)1522-2675(19990804)82:8<1211::AID-HLCA1211>3.0.CO;2-K](https://doi.org/10.1002/(SICI)1522-2675(19990804)82:8%3c1211::AID-HLCA1211%3e3.0.CO;2-K)
4. H.S. Boudier,G. Smeets, G Brouwer, J. van Rossum. Central and peripheral alpha adrenergic activity of imi­dazoline derivatives. *Life Sciences* **15** (1974) 887-899. <https://doi.org/10.1016/0024-3205(74)90005-8>
5. S. Sanli, B. Akmese, Y. Altun. Determination of pKa values of some antipsychotic drugs by HPLC--correlations with the Kamlet and taft solvatochromic parameters and HPLC analysis in dosage forms. *Journal of AOAC International* **96** (2013) 60-66. <https://doi.org/10.5740/jaoacint.11-494>.
6. A. Arra, S. Fernández de Betoño, J. M. Moreda, A. Cid, J. F. Arranz. Cathodic stripping voltammetric determination of doxazosin in urine and pharmaceutical tablets using carbon paste electrodes. *Analyst* **122** (1997) 849-854. <https://doi.org/10.1039/a701210a>.
7. P. Wiczling, A. Nasal, Ł. Kubik, R. Kaliszan. A new pH/organic modifier gradient RP HPLC method for convenient determination of lipophilicity and acidity of drugs as applied to established imidazoline agents*. European Journal of Pharmaceutical Sciences* **47** (2012) 1-5. <https://doi.org/10.1016/j.ejps.2012.04.021>
8. K.Y. Tama, K. Takács-Novák. Multi-wavelength spectrophotometric determination of acid dissociation constants: a validation study. *Analytica Chimica Acta* **434** (2001) 157-167. <https://doi.org/10.1016/S0003-2670(01)00810-8>
9. T. Douglas, R. K. Sharma, J. F. Walmsley, R. C.Hider. Ionization processes of some harmala alkaloids. *Molecular Pharmacology* **23** (1983) 614-618.
10. T. J. DiFeo, J. E. Shuster. Indapamide. *Analytical Profiles of Drug Substancesa and Excipients* **23** (1994) 229-268. <https://doi.org/10.1016/S0099-5428(08)60604-8>
11. R. Ruiz, C. Ràfols, M. Rosés, E. Bosch. A potentially simpler approach to measure aqueous pKa of insoluble basic drugs containing amino groups. *Journal of Pharmaceutical Sciences* **92** (2003) 1473-1481. doi: 10.1002/jps.10415.
12. J. Kelder, C. Funke, T. De Boer, L. Delbressine, D. Leysen, V. Nickolson. A comparison of the physicochemical and biological properties of mirtazapine and mianserin*. Journal of Pharmacy and Pharmacology* **49** (1997) 403-411. <https://doi.org/10.1111/j.2042-7158.1997.tb06814.x>
13. M. Meloun, T. Syrový, A. Vrána. The thermodynamic dissociation constants of ambroxol, antazoline, naphazoline, oxymetazoline and ranitidine by the regression analysis of spectrophotometric data. *Talanta* **62** (2004) 511-522. <https://doi.org/10.1016/j.talanta.2003.08.027>
14. M. Meloun, T. Syrový, A. Vrána. The thermodynamic dissociation constants of losartan, paracetamol, phenylephrine and quinine by the regression analysis of spectrophotometric data. *Analytica Chimica Acta* **533** (2005) 97-110. <https://doi.org/10.1016/j.aca.2004.11.007>
15. U. Domańska, A. Pobudkowska, A Pelczarska, L. Zukowski. Modelling, solubility and pK(a) of five sparingly soluble drugs. *International Journal of Pharmaceutics* **403** (2011) 115-122. <https://doi.org/10.1016/j.ijpharm.2010.10.034>
